# Targeted degradation of CDK4/6 by LA-CB1 inhibits EMT and suppresses tumor growth in orthotopic breast cancer

**DOI:** 10.1038/s41598-025-92494-8

**Published:** 2025-03-04

**Authors:** Jingliang He, Shunfang Liu, Siyi Zhang, Qi Gao, Lan Zhu, Ningyang Xu, Zhongke Hu, Xingyu Zhang, Shaojie Ma, Xiujun Wang, Bin Liu, Wei Liu

**Affiliations:** 1 Jiangsu Key Laboratory of Marine Pharmaceutical Compound Screening, College of Pharmacy, Jiangsu Ocean University, Lianyungang, 222005 China; 2 Department of Oncology, Tongji Hospital of Tongji Medical College, Huazhong University of Science and Technology, Jiefang Road 1095, Wuhan, 430030 China; 3 Cancer Center and Department of Pharmacology and Toxicology, Medical College of Wisconsin, Milwaukee, WI 53226 USA

**Keywords:** CDK4/6 degradation, LA-CB1, Cell cycle arrest, Breast cancer, Ubiquitin-proteasome pathway, Epithelial-mesenchymal transition (EMT), Biochemistry, Cancer, Chemical biology, Oncology

## Abstract

Cyclin-dependent kinases 4 and 6 (CDK4/6) are central regulators of cell cycle progression and frequently dysregulated in cancers, including breast cancer. While selective CDK4/6 inhibitors like Palbociclib, Ribociclib, and Abemaciclib have shown clinical benefit in hormone receptor-positive (HR+) breast cancer, their efficacy is often limited by resistance mechanisms and dose-limiting toxicities. In this study, we developed LA-CB1, a novel Abemaciclib derivative that induces CDK4/6 degradation through the ubiquitin-proteasome pathway, aiming to achieve sustained inhibition of the CDK4/6-Rb axis. LA-CB1 demonstrated potent anti-proliferative effects in various breast cancer cell lines, with notable efficacy in triple-negative breast cancer (TNBC) and HR + breast cancer models. Molecular docking studies confirmed high-affinity binding of LA-CB1 to the ATP-binding pocket of CDK4/6. Mechanistic studies revealed that LA-CB1 induces G0/G1 cell cycle arrest and promotes apoptosis through the degradation of CDK4/6. Importantly, LA-CB1 also suppressed epithelial-mesenchymal transition (EMT), inhibiting key processes such as cell migration, invasion, and angiogenesis, indicating its ability to disrupt multiple hallmarks of cancer. In an orthotopic breast cancer model, LA-CB1 significantly reduced tumor growth in a dose-dependent manner. These results suggest that LA-CB1 represents a promising therapeutic strategy by targeting CDK4/6 for degradation, addressing limitations associated with current CDK4/6 inhibitors, and providing broad anti-tumor activity in aggressive cancer types like TNBC.

**Supplementary Information:**

The online version contains supplementary material available at 10.1038/s41598-025-92494-8.

## Introduction

Cyclin-dependent kinases 4 and 6 (CDK4/6) play a crucial role in regulating cell cycle progression, specifically in driving the transition from the G1 to S phase by phosphorylating the retinoblastoma (Rb) protein^1–3^. This phosphorylation event releases E2F transcription factors, which then activate the transcription of genes required for DNA replication and cell cycle continuation^4^. Dysregulation of the CDK4/6-Rb-E2F pathway is a common feature in various cancers, particularly in breast cancer, where CDK4/6 overactivation leads to uncontrolled cell proliferation and tumor growth^5,6^. As key drivers of tumor progression, CDK4/6 have become important therapeutic targets^7^.

The development of selective CDK4/6 inhibitors, such as Palbociclib, Ribociclib, and Abemaciclib, has significantly changed the treatment approach for hormone receptor-positive (HR+) breast cancer^8–10^. These drugs have been shown to extend progression-free survival and improve clinical outcomes by inhibiting the ATP-binding sites of CDK4/6, preventing Rb phosphorylation, and arresting the cell cycle in G1 phase^11,12^. However, the success of CDK4/6 inhibitors is often compromised by the emergence of resistance. Mechanisms of resistance include Rb mutations that reduce sensitivity to CDK4/6 inhibition, and compensatory activation of other kinases such as CDK2, which can bypass the inhibition of CDK4/6^13,14^. Furthermore, long-term use of these inhibitors is associated with side effects like neutropenia, limiting treatment duration and dosage^15^.

In light of these challenges, new therapeutic approaches are necessary to more effectively target cancers driven by CDK4/6 and overcome resistance^16^. One promising approach involves developing compounds that degrade CDK4/6 proteins rather than just inhibiting their activity^17^. A notable strategy in this field is PROTAC (Proteolysis Targeting Chimeras) technology, which utilizes the ubiquitin-proteasome system to degrade specific proteins^18,19^. By promoting proteasomal degradation of CDK4/6, these compounds eliminate the proteins, potentially achieving more sustained suppression of the cell cycle^20,21^. Moreover, this approach could help prevent the compensatory signaling pathways that contribute to resistance^22^. Although PROTACs offer advantages over traditional inhibitors, their large molecular size can pose challenges for drug development, including issues with bioavailability^23,24^.

In this study, we present LA-CB1, a new derivative of Abemaciclib that induces degradation of CDK4/6 through the ubiquitin-proteasome pathway. Unlike conventional CDK4/6 inhibitors, which block enzymatic activity, LA-CB1 promotes degradation of these proteins, offering a different therapeutic approach that may address resistance and toxicity challenges. Given the critical role of CDK4/6 in HR + breast cancer and their increasing relevance in aggressive cancers such as triple-negative breast cancer (TNBC), we evaluated LA-CB1’s therapeutic potential in multiple cancer models. Through a series of in vitro and in vivo studies, we aimed to explore whether LA-CB1 could serve as a potent CDK4/6 degrader with enhanced anti-cancer effects, laying the groundwork for its further development in cancers driven by CDK4/6 signaling, particularly in challenging subtypes like TNBC.

## Materials and methods

### Synthesis of LA-CB1

LA-CB1 (2-((2-chloro-4-((4-(4-fluoro-1-isopropyl-2-methyl-1 H-benzo[d]imidazol-6-yl)pyrimidin-2-yl)amino)phenoxy)methyl)-3-cyanopyridine) was synthesized via a palladium-catalyzed cross-coupling reaction. Briefly, 2-((4-amino-2-chlorophenoxy)methyl)-3-cyanopyridine (1.0 mmol) and 6-(2-chloropyrimidin-4-yl)-4-fluoro-1-isopropyl-2-methyl-1 H-benz[d]imidazole (1.1 mmol) were dissolved in 1,4-dioxane (10 mL) under nitrogen atmosphere. Palladium acetate (Pd(OAc)₂) (0.05 mmol), Cs₂CO₃ (2.0 mmol), and Xantphos (0.1 mmol) were added, and the reaction mixture was refluxed at 110 °C for 24 h. Upon completion, the reaction mixture was cooled to room temperature, concentrated under reduced pressure, and purified by silica gel chromatography using ethyl acetate/hexane as the eluent. The product was obtained as a yellow solid (yield: 53.98%). The structure of LA-CB1 was confirmed by ¹H NMR, ¹³C NMR, and HRMS.

### Cell lines and culture conditions

Human breast cancer cell lines MDA-MB-231 and MCF-7, human cervical cancer cell line HeLa, human renal cell carcinoma cell line A498, and human colon cancer cell line HCT116 were obtained from the American Type Culture Collection (ATCC). Cells were cultured in Dulbecco’s Modified Eagle Medium (DMEM) supplemented with 10% fetal bovine serum (FBS), 100 U/mL penicillin, and 100 µg/mL streptomycin at 37 °C in a humidified incubator with 5% CO₂. Cells were passaged every 2–3 days using 0.25% trypsin-EDTA.

### MTT assay

Cell viability was assessed using the MTT assay. Briefly, cells were seeded into 96-well plates at a density of 5 × 10³ cells/well and allowed to adhere overnight. Cells were treated with varying concentrations of LA-CB1, Abemaciclib, Ribociclib, or Imatinib for 48 h. After treatment, MTT reagent (5 mg/mL) was added to each well and incubated for an additional 4 h at 37 °C. The formazan crystals formed were dissolved in DMSO, and absorbance was measured at 570 nm using a microplate reader (Bio-Rad). IC₅₀ values were calculated using GraphPad Prism software, (version 10, available at https://www.graphpad.com/scientific-software/prism/).

### Colony formation assay

For the colony formation assay, MDA-MB-231 and MCF-7 cells were seeded in 6-well plates at a density of 500 cells/well and treated with LA-CB1 (0.25, 0.5, 1 µM) or control for 24 h. After treatment, the medium was replaced, and cells were allowed to grow for 10–14 days. Colonies were fixed with 4% paraformaldehyde and stained with 0.5% crystal violet. Colonies containing more than 50 cells were counted using ImageJ software, (version 1.53, available at https://imagej.nih.gov/ij/). The experiment was performed in triplicate.

### EdU incorporation assay

DNA synthesis was evaluated using the 5-ethynyl-2′-deoxyuridine (EdU) incorporation assay. MDA-MB-231 and MCF-7 cells were seeded in 24-well plates at 2 × 10⁴ cells/well and treated with LA-CB1 or controls for 48 h. Cells were incubated with 10 µM EdU for 2 h, fixed with 4% paraformaldehyde, and stained with the Click-iT EdU Imaging Kit (Invitrogen) according to the manufacturer’s instructions. Nuclei were counterstained with Hoechst 33,342. Images were captured using a fluorescence microscope (Olympus IX71), and EdU-positive cells were quantified using ImageJ.

### Wound healing assay

Cell migration was assessed using the wound healing assay. MDA-MB-231 and MCF-7 cells were seeded in 6-well plates and grown to confluence. A uniform scratch was created using a 200 µL pipette tip, and cells were washed with PBS to remove debris. Cells were then treated with LA-CB1 or control in serum-free medium. Images of the wound area were captured at 0, 24, and 48 h using an Olympus IX71 microscope, and the migration distance was quantified using ImageJ software. Wound closure was calculated as the percentage of the original wound area.

### Transwell invasion assay

Cell invasion was evaluated using Transwell chambers with 8 μm pores (Corning) coated with Matrigel (BD Biosciences)^25^. MDA-MB-231 and MCF-7 cells were resuspended in serum-free medium and seeded into the upper chamber at a density of 1 × 10⁵ cells/well. The lower chamber was filled with DMEM containing 10% FBS. After 24 h, non-invading cells were removed from the upper chamber, and invading cells on the lower surface were fixed with methanol and stained with 0.1% crystal violet. Images were captured using an Olympus IX71 microscope, and invading cells were quantified using ImageJ software.

### The ARRIVE statement

The study was conducted in compliance with the ARRIVE guidelines (Animal Research: Reporting of In Vivo Experiments). All animal experiments, including the Chicken Chorioallantoic Membrane (CAM) assay and the in vivo orthotopic mice tumor model assay, were performed in accordance with relevant institutional guidelines and regulations, and were approved by the Ethics Committee of Jiangsu Ocean University. Efforts were made to minimize suffering and reduce the number of animals used in accordance with the 3Rs principle (Replacement, Reduction, and Refinement).

### CAM assay

The anti-angiogenic effects of LA-CB1 were evaluated using the CAM assay^26^. Fertilized chicken eggs were obtained from the Wufengshan Chicken Farm in Tongling, Anhui Province, China, and incubated at 37.5 °C with 60% humidity. The gestation period of the eggs used in our research was 21 days. Fertilized chicken eggs were incubated at 37.5 °C with 60% humidity. On day 7 of incubation, a small hole was created in the shell, and MDA-MB-231 or MCF-7 cells (1 × 10^6^ cells) were inoculated onto the CAM. After 48 h, LA-CB1 or control was applied topically onto the tumor site. On day 11, tumors were excised, photographed, and weighed. The vascular density surrounding the tumor was analyzed using ImageJ software.

### Flow cytometry for apoptosis and cell cycle analysis

Apoptosis was assessed using the Annexin V-FITC/PI Apoptosis Detection Kit (BD Biosciences) according to the manufacturer’s protocol. MDA-MB-231 and MCF-7 cells were treated with LA-CB1 (0.25, 0.5, 1 µM) for 48 h, stained with Annexin V-FITC and propidium iodide (PI), and analyzed by flow cytometry (BD FACSCanto II). Data were analyzed using FlowJo software, (version 10.8, available at https://www.flowjo.com/). For cell cycle analysis, cells were fixed in 70% ethanol at -20 °C for 24 h, stained with PI/RNase solution (BD Biosciences), and analyzed by flow cytometry. Cell cycle distribution was determined using ModFit LT software, (version 5.0, available at https://www.veritysoftware.com/modfit/).

### Western blot analysis

Total protein was extracted from MDA-MB-231 and MCF-7 cells treated with LA-CB1 or control using RIPA buffer containing protease and phosphatase inhibitors. Protein concentration was determined using the BCA Protein Assay Kit (Thermo Scientific). Equal amounts of protein were separated by SDS-PAGE and transferred to PVDF membranes. Membranes were blocked with 5% non-fat milk and incubated overnight at 4 °C with primary antibodies against CDK4, CDK6, Cyclin D1, E2F1, Rb, P-Rb, cleaved caspase-3, and GAPDH (all from Cell Signaling Technology). After incubation with HRP-conjugated secondary antibodies, bands were visualized using ECL reagent (Bio-Rad) and quantified by ImageJ software. The uncropped full Western blot images, including all three biological replicates, are provided in the supplementary materials for full transparency and validation of the results.

### RNA sequencing

The cells were processed using TRIzol reagent (Invitrogen Company, USA) to extract RNA, which was then sent to Shanghai Sangon Technology Co., LTD for RNA sequencing (RNA-Seq). The sequencing data obtained were subsequently analyzed using bioinformatics techniques to derive meaningful insights^27^. The sequencing data were visualized using R software, (version 4.2.3, available at https://www.r-project.org/).

### In vivo orthotopic tumor model

ICR mice (6 weeks old, female) were orthotopically injected with 4T1-LUC cells (1 × 10⁶ cells) into the mammary fat pad to establish breast tumors. Prior to injection, all mice were anesthetized using isoflurane, and anesthesia was maintained throughout the procedure. After the completion of the experiment, the mice were euthanized using cervical dislocation, in accordance with ethical guidelines. Mice were randomized into groups and treated with LA-CB1 (0.5 mg/kg, 1 mg/kg, 2 mg/kg) or vehicle control via intraperitoneal injection every other day. Tumor growth was monitored by bioluminescence imaging using the IVIS Spectrum Imaging System (PerkinElmer) at days 1, 11, and 21 post-tumor inoculation. Fluorescence intensity was quantified as photons/second using Living Image software.

### Statistical analysis

All experiments were performed in triplicate unless otherwise stated. Data are presented as mean ± SD. Statistical significance was determined using one-way ANOVA followed by Tukey’s post hoc test or two-way ANOVA for multi-group comparisons. Differences were considered statistically significant at *p* < 0.05. All statistical analyses were performed using GraphPad Prism software.

## Results

### Synthesis and biological evaluation of LA-CB1

**Fig. 1:**
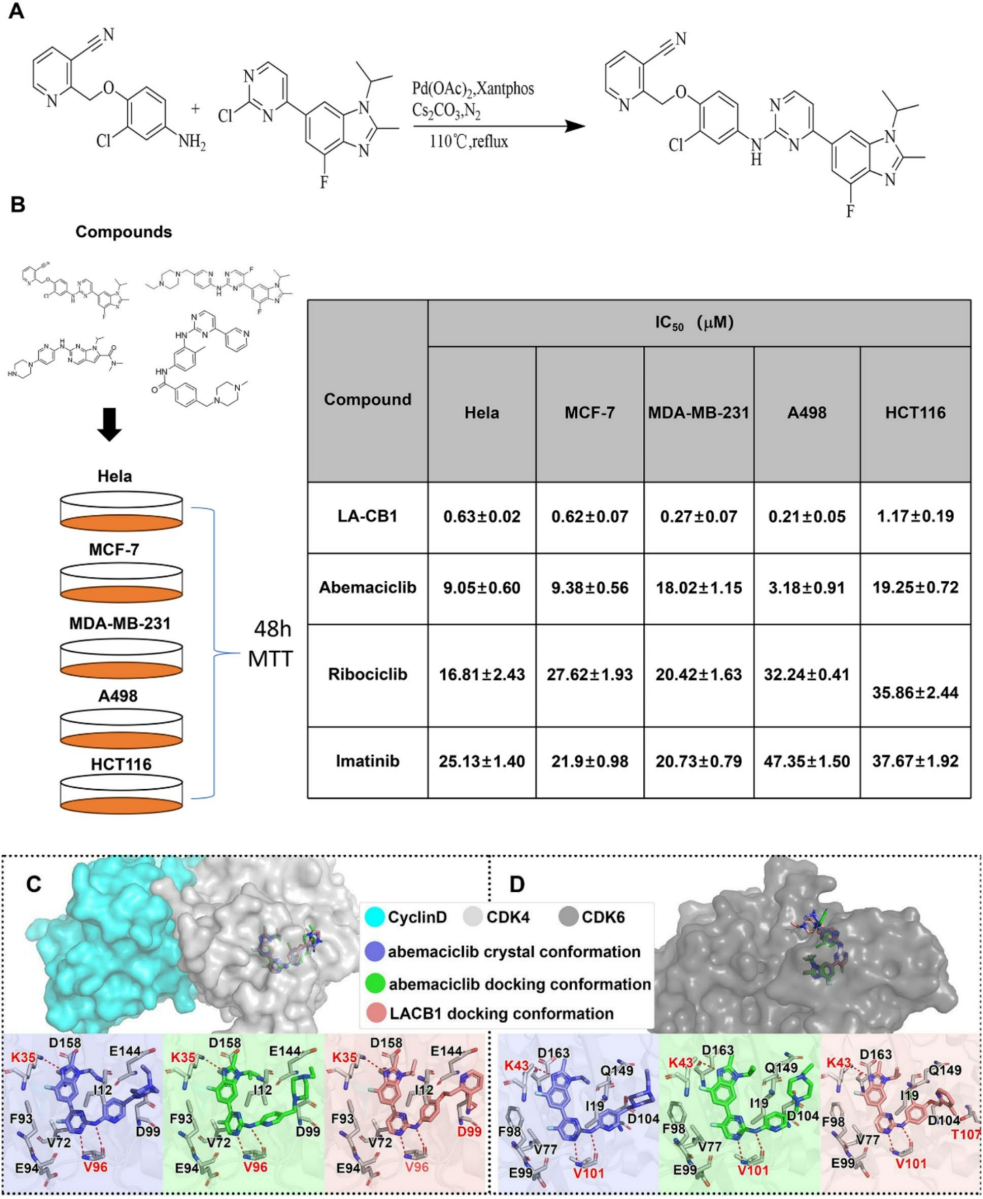
Synthesis and Antiproliferative Activity of LA-CB1 in Cancer Cell Lines. (**A**) Chemical structure and synthesis of LA-CB1. The compound LA-CB1 was synthesized through a palladium-catalyzed cross-coupling reaction between 2-((4-amino-2-chlorophenoxy)methyl)-3-cyanopyridine and 6-(2-chloropyrimidin-4-yl)-4-fluoro-1-isopropyl-2-methyl-1 H-benz[d]imidazole, yielding the final product. (**B**) IC50 values of LA-CB1 in a panel of cancer cell lines as determined by the MTT assay after 48 h of treatment. LA-CB1 displayed potent antiproliferative activity across all tested cell lines, with the lowest IC50 values in A498 and MDA-MB-231 cells. Data are shown as mean ± SD from three independent experiments. (**C, D**) Molecular docking of LA-CB1 and Abemaciclib with CDK4 and CDK6. (**C**) Docking conformation of LA-CB1 (green) compared with Abemaciclib (blue) in the ATP-binding site of CDK6. (**D**) Hydrogen bonding interactions between LA-CB1 and the residues in the CDK6 binding pocket are highlighted. LA-CB1 forms an additional hydrogen bond via its cyano group, contributing to its enhanced binding affinity.

The novel compound LA-CB1, 2-((2-chloro-4-((4-(4-fluoro-1-isopropyl-2-methyl-1 H-benzo[d]imidazol-6-yl)pyrimidin-2-yl)amino)phenoxy)methyl)-3-cyanopyridine, was synthesized through a palladium-catalyzed cross-coupling reaction (Fig. 1A). The reaction was carried out between 2-((4-amino-2-chlorophenoxy)methyl)-3-cyanopyridine and 6-(2-chloropyrimidin-4-yl)-4-fluoro-1-isopropyl-2-methyl-1 H-benz[d]imidazole in the presence of Pd(OAc)_2_, Xantphos, and Cs_2_CO_3_, with 1,4-dioxane as the solvent, at 110 °C under reflux conditions. This reaction yielded LA-CB1 as a yellow solid with a yield of 53.98%. The structure of LA-CB1 was confirmed through^1^H NMR^13^, C NMR, and HRMS analysis, ensuring the correct chemical identity of the compound (Supplementary Fig. 1A-B). The antiproliferative activity of LA-CB1 was evaluated using the MTT assay in a panel of cancer cell lines, including Hela, MCF-7, MDA-MB-231, A498, and HCT116 (Fig. 1B). After 48 h of treatment, LA-CB1 demonstrated potent inhibitory effects, with IC_50_ values ranging from 0.21 µM in A498 cells to 1.17 µM in HCT116 cells. Among the tested cell lines, MDA-MB-231 and A498 showed particularly high sensitivity to LA-CB1, with IC_50_ values of 0.27 µM and 0.21 µM, respectively. In comparison, the reference CDK inhibitors Abemaciclib, Ribociclib, and Imatinib displayed significantly higher IC_50_ values in MDA-MB-231 and A498 cells. For example, the IC_50_ of Abemaciclib in MDA-MB-231 cells was 18.02 µM, that of Ribociclib was 27.62 µM, and Imatinib had an IC50 of 20.73 µM, which were all markedly higher than LA-CB1’s IC_50_ of 0.27 µM, highlighting the superior efficacy of LA-CB1 in inhibiting cell proliferation (Fig. 1B). The antiproliferative activity of LA-CB1 in normal cells was significantly weaker than that of MDA-MB231, For example, in 293T cells the IC_50_ was 8.92 µM (Supplementary Fig. 2A). The results suggest that LA-CB1 has less negative effects on normal cells when used at low doses. However, for long-term or high-dose applications, further studies on its potential side effects are still needed. The IC_50_ for LA-CB1 in the independent triple-negative breast cancer cell line MDA-MB-468 was 0.45 µM (Supplementary Fig. 2B). To gain insights into the molecular interactions between LA-CB1 and its targets, molecular docking studies were performed to compare the binding affinity of LA-CB1 and Abemaciclib towards CDK4 and CDK6 (Fig. 1C-D). The docking scores for LA-CB1 were − 9.37 kcal/mol for CDK4 and − 8.95 kcal/mol for CDK6, which were slightly better than the docking scores for Abemaciclib (-9.01 kcal/mol for CDK4 and − 8.52 kcal/mol for CDK6). Structural analysis revealed that LA-CB1 shares a similar binding conformation with Abemaciclib, as both compounds bind in the ATP-binding pocket of CDK4/6. However, the presence of a cyano group in LA-CB1 allowed for the formation of an additional hydrogen bond with the kinase active site, potentially explaining its enhanced binding affinity compared to Abemaciclib. This additional hydrogen bond may also contribute to LA-CB1’s superior antiproliferative activity across multiple cancer cell lines.

### LA-CB1 inhibits the clonogenic growth and DNA synthesis in breast Cancer cells

**Fig. 2:**
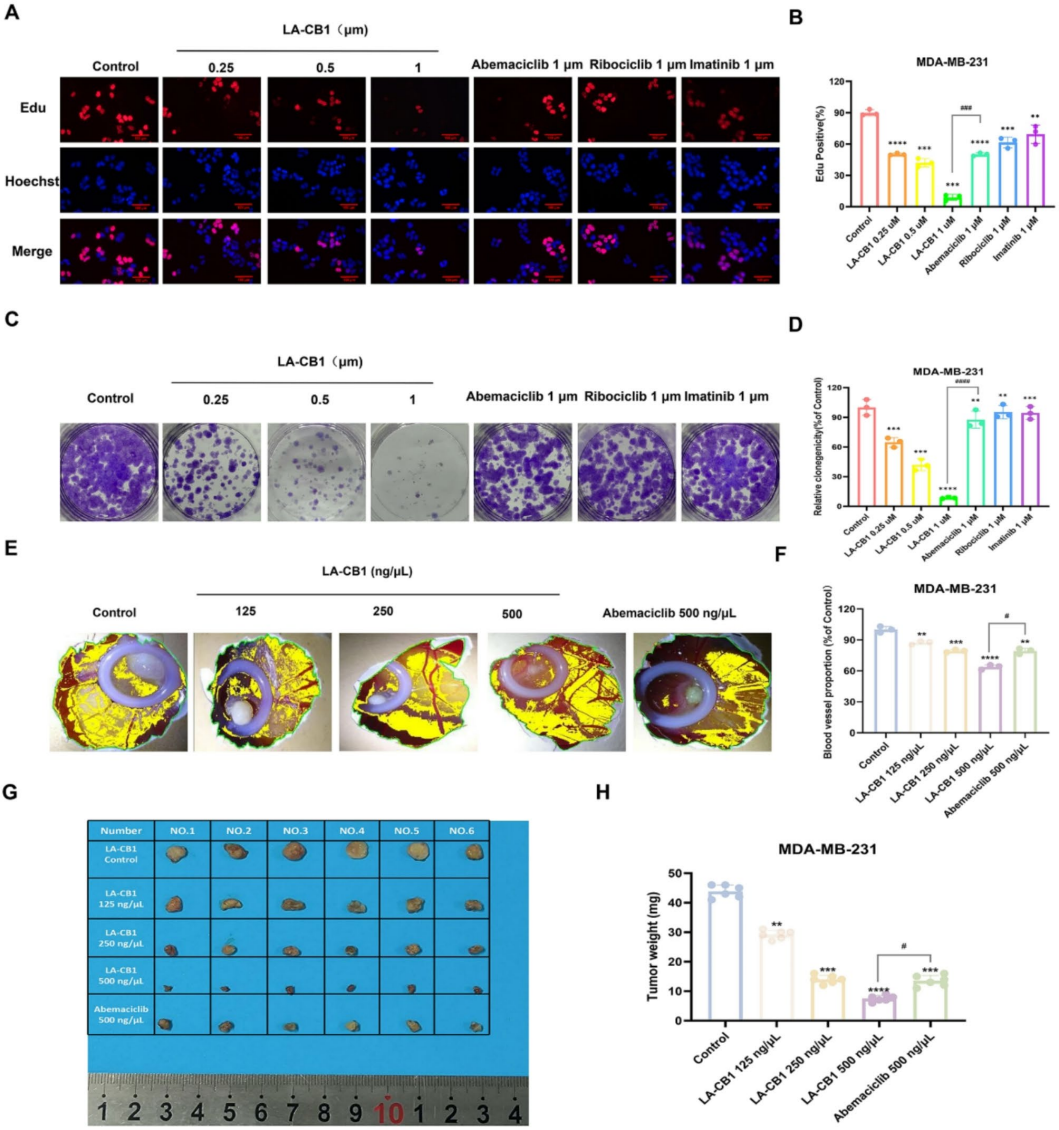
LA-CB1 Inhibits DNA Synthesis and Clonogenic Growth in MDA-MB-231 Cells, and Suppresses Angiogenesis in CAM Models. (**A**) EdU incorporation assay in MDA-MB-231 cells. Cells were treated with increasing concentrations of LA-CB1 (0.25, 0.5, 1 µM) for 48 h, followed by EdU staining. Representative images show EdU-positive cells (red) and Hoechst-stained nuclei (blue). Scale bar, 50 μm. (**B**) Quantification of EdU-positive cells in MDA-MB-231 cells. LA-CB1 significantly reduced EdU incorporation in a dose-dependent manner. Data are represented as mean ± SD (*n* = 3). *p* < 0.001 compared to control. (**C**) Colony formation assay in MDA-MB-231 cells treated with LA-CB1 for 10 days. Representative images of colonies stained with crystal violet. (**D**) Quantification of relative colony numbers in MDA-MB-231 cells. LA-CB1 markedly reduced colony formation, with 1 µM LA-CB1 inhibiting clonogenic survival by more than 80%. Data are shown as mean ± SD (*n* = 3). *p* < 0.001 compared to control. (**E**) Representative images of CAM assays showing tumor size and vascularization in MDA-MB-231 tumor models treated with increasing concentrations of LA-CB1. (**F**) Quantification of basal vascular proportion surrounding the tumors in the CAM assay. LA-CB1 significantly inhibited angiogenesis in a dose-dependent manner, with effects comparable to Abemaciclib. Data are represented as mean ± SD (*n* = 3). *p* < 0.01 compared to control. (**G, H**) Measurement of tumor weight in MDA-MB-231 tumors excised from CAM models. LA-CB1 reduced tumor weight in a dose-dependent manner. *p* < 0.001 compared to control.

To further investigate the antiproliferative effects of LA-CB1, its impact on DNA synthesis was examined using EdU incorporation assays in both MDA-MB-231 and MCF-7 breast cancer cells. Treatment with LA-CB1 at concentrations of 0.25, 0.5, and 1 µM significantly reduced the number of EdU-positive cells in both cell lines, indicating that LA-CB1 inhibits DNA synthesis in a dose-dependent manner (Fig. 2A-B). In MDA-MB-231 cells, 1 µM LA-CB1 reduced EdU incorporation by approximately 80%, a level of inhibition comparable to Abemaciclib, Ribociclib, and Imatinib. In MCF-7 cells, LA-CB1 at 1 µM decreased EdU incorporation by approximately 75%, demonstrating a similar efficacy to Abemaciclib and Ribociclib, while Imatinib did not produce a significant effect (Supplementary Fig. 3A-B, *p* < 0.001 for LA-CB1 compared to control in both cell lines). To further assess the effects of LA-CB1 on the clonogenic survival of cancer cells, colony formation assays were performed in MDA-MB-231 and MCF-7 cells. In MDA-MB-231 cells, treatment with 1 µM LA-CB1 almost completely abolished colony formation, reducing the number of colonies by more than 85%, significantly outperforming the effects of Abemaciclib, Ribociclib, and Imatinib (Fig. 2C-D, *p* < 0.001). In MCF-7 cells, 1 µM LA-CB1 reduced colony formation by over 80%, again showing greater efficacy than Ribociclib and Imatinib, and demonstrating comparable potency to Abemaciclib (Supplementary Fig. 3C-D, *p* < 0.001). The anti-angiogenic properties of LA-CB1 were examined using the chicken chorioallantoic membrane (CAM) assay in both MDA-MB-231 and MCF-7 tumor models. Tumors treated with LA-CB1 at concentrations of 125, 250, and 500 ng/µL displayed a dose-dependent reduction in basal vascular proportion, indicating that LA-CB1 effectively inhibits angiogenesis. In the MDA-MB-231 model, treatment with 500 ng/µL LA-CB1 significantly reduced angiogenesis, producing effects comparable to those observed with 500 ng/µL Abemaciclib (Fig. 2E-F). Similarly, in MCF-7 tumors, 500 ng/µL LA-CB1 demonstrated strong anti-angiogenic activity, showing a reduction in angiogenesis comparable to that of 500 ng/µL Abemaciclib (Supplementary Fig. 3E-F). Furthermore, LA-CB1 treatment significantly reduced tumor weight in the CAM model for both MDA-MB-231 and MCF-7 xenografts. In the MDA-MB-231 model, treatment with 500 ng/µL LA-CB1 resulted in a reduction of tumor weight by more than 70%, a decrease comparable to the effects of Abemaciclib (Fig. 2G-H). Similarly, in the MCF-7 model, LA-CB1 at 500 ng/µL produced a reduction in tumor weight that was comparable to Abemaciclib, further confirming the potent anti-tumor effects of LA-CB1 (Supplementary Fig. 3G-H). These results demonstrate that LA-CB1 exhibits significant anti-angiogenic and anti-tumor properties in vivo, suggesting its potential as a novel therapeutic agent for the treatment of cancer.

### LA-CB1 inhibits migration and invasion in MDA-MB-231 and MCF-7 cells

**Fig. 3:**
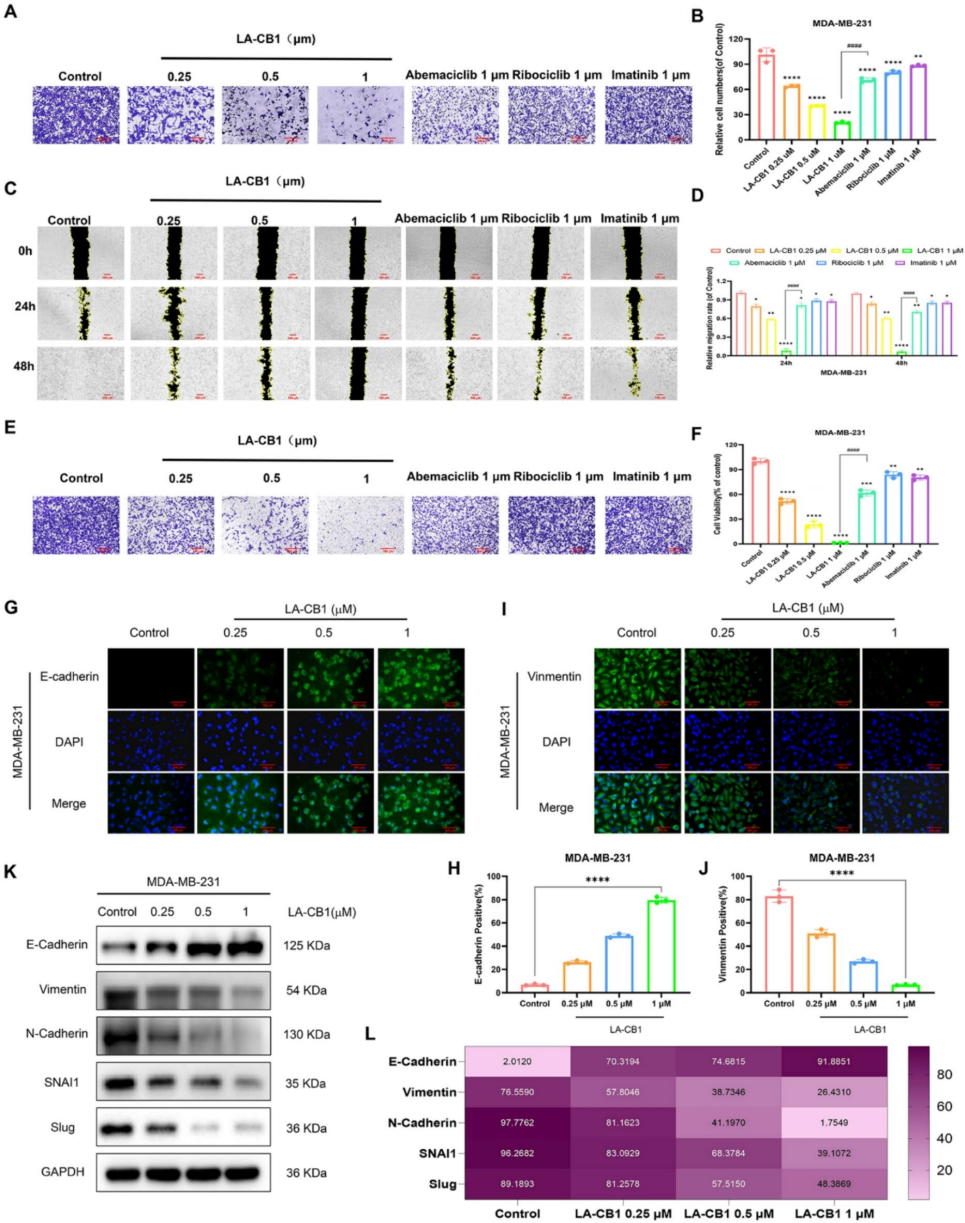
LA-CB1 Inhibits Cell Adhesion, Migration, and Invasion in MDA-MB-231 Cells, and Modulates EMT Markers. (**A**) Representative images of cell adhesion assays in MDA-MB-231 cells treated with LA-CB1 (0.25, 0.5, 1 µM) for 2 h. Adherent cells were stained with crystal violet. (**B**) Quantification of adherent cells following LA-CB1 treatment. LA-CB1 significantly inhibited adhesion in a dose-dependent manner. Data are shown as mean ± SD (*n* = 3). *p* < 0.001 compared to control. (**C**) Wound healing assay in MDA-MB-231 cells treated with increasing concentrations of LA-CB1. Representative images show wound closure at 0, 24, and 48 h after treatment. (**D**) Quantification of wound closure after 24 and 48 h of treatment. LA-CB1 significantly delayed wound closure in MDA-MB-231 cells. Data are represented as mean ± SD (*n* = 3). *p* < 0.001 compared to control. (**E**) Transwell invasion assay showing MDA-MB-231 cells treated with LA-CB1 for 24 h. Representative images of invaded cells stained with crystal violet. (**F**) Quantification of invaded cells in the Transwell assay. LA-CB1 significantly reduced cell invasion in a dose-dependent manner. Data are shown as mean ± SD (*n* = 3). *p* < 0.001 compared to control. (**G-J**) Immunofluorescence staining of E-cadherin (G) and Vimentin (H) in MDA-MB-231 cells treated with increasing concentrations of LA-CB1 for 48 h. Scale bar, 100 μm. Data are shown as mean ± SD (*n* = 3). *p* < 0.001 compared to control. (**K, L**) Western blot analysis and quantification of E-cadherin, Vimentin, N-cadherin, SNAI1, and Slug expression levels following LA-CB1 treatment. LA-CB1 treatment led to a dose-dependent increase in E-cadherin and decrease in mesenchymal markers in MDA-MB-231 cells. Quantification data are shown as mean ± SD from three independent experiments. *p* < 0.01 compared to control.

The effect of LA-CB1 on cell adhesion was evaluated in both MDA-MB-231 and MCF-7 cells using adhesion assays. In MDA-MB-231 cells, LA-CB1 treatment significantly inhibited cell adhesion in a dose-dependent manner. At 1 µM, LA-CB1 reduced cell adhesion by over 80%, a more pronounced effect than those observed with Abemaciclib, Ribociclib, and Imatinib (Fig. 3A-B). Mitomycin C is a chemical that inhibits cell division and is widely used in cell proliferation studies. It inhibits cell proliferation in experiments by cross-linking DNA and preventing cells from entering the division phase. In MDA-MB-231 cells, we performed a scratch assay using DMSO, mitomycin C, LA-CB1, or a combination thereof to eliminate the potential interference of cell proliferation on migration. The experimental results suggest that the LA-CB1-induced inhibition of wound closure is not due to cell cycle arrest (Supplementary Fig. 4A-B). We performed wound healing and Transwell invasion assays in another TNBC cell line, MDA-MB-468. The results further validated the findings obtained in MDA-MB-231 cells, indicating a dose-dependent inhibitory effect of LA-CB1 on migration and invasion (Supplementary Fig. 4C-F). Similarly, in MCF-7 cells, LA-CB1 at 1 µM reduced adhesion by more than 75%, showing comparable efficacy to Abemaciclib, and greater efficacy than Ribociclib and Imatinib (Supplementary Fig. 5A-B). The impact of LA-CB1 on cell migration was assessed using a wound healing assay. In MDA-MB-231 cells, LA-CB1 treatment significantly inhibited wound closure in a dose-dependent manner, with 1 µM LA-CB1 almost completely preventing wound closure after 48 h (Fig. 3C-D). In comparison, Abemaciclib and Ribociclib also delayed wound healing but were less effective than LA-CB1. Similarly, in MCF-7 cells, LA-CB1 reduced cell migration by more than 70% at 1 µM, exhibiting comparable efficacy to Abemaciclib, and superior efficacy to Ribociclib and Imatinib (Supplementary Fig. 5C-D). The effect of LA-CB1 on cell invasion was further investigated using transwell invasion assays. In MDA-MB-231 cells, LA-CB1 significantly inhibited invasion in a dose-dependent manner, with 1 µM LA-CB1 reducing cell invasion by over 85% (Fig. 3E-F), a stronger effect than those observed with Abemaciclib, Ribociclib, or Imatinib. Similarly, in MCF-7 cells, LA-CB1 at 1 µM reduced invasion by more than 80%, demonstrating similar efficacy to Abemaciclib, and outperforming both Ribociclib and Imatinib (Supplementary Fig. 5E-F). To explore the molecular mechanisms underlying LA-CB1’s inhibitory effects on cell adhesion, migration, and invasion, immunofluorescence and Western blot analyses were performed to assess the expression of key epithelial-mesenchymal transition (EMT) markers^28^. In MDA-MB-231 cells, LA-CB1 treatment upregulated E-cadherin, an epithelial marker, and downregulated the mesenchymal markers Vimentin, N-cadherin, SNAI1, and Slug, in a dose-dependent manner (Fig. 3G-L). Similar changes were observed in MCF-7 cells, where LA-CB1 treatment resulted in increased expression of E-cadherin and decreased levels of Vimentin, N-cadherin, SNAI1, and Slug (Supplementary Fig. 5G-L). Quantitative analysis of Western blot results confirmed these observations, showing a significant upregulation of E-cadherin and a marked downregulation of mesenchymal markers in both MDA-MB-231 and MCF-7 cells following treatment with LA-CB1 (Fig. 3K-L, Supplementary Fig. 5K-L). These findings suggest that LA-CB1 inhibits cancer cell adhesion, migration, and invasion by modulating EMT-related proteins, further supporting its potential as an effective anti-metastatic agent.

### LA-CB1 inhibits EMT, cell cycle progression, and DNA damage response

**Fig. 4:**
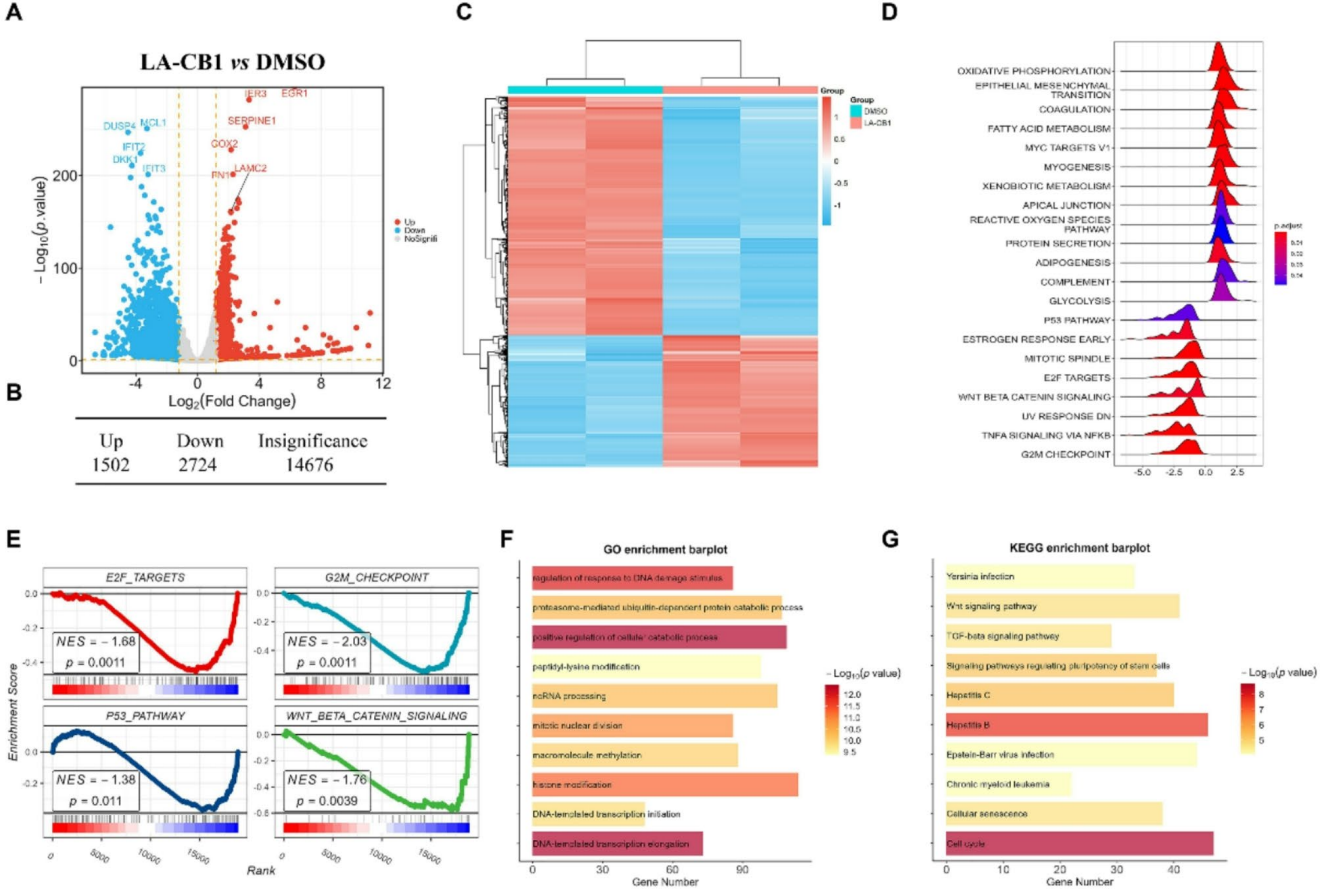
LA-CB1 Modulates EMT, Cell Cycle Progression, and DNA Damage Response Pathways in Cancer Cells. (**A**) Volcano plot showing differentially expressed genes (DEGs) in LA-CB1-treated vs. DMSO-treated MDA-MB-231 cells. Significantly upregulated and downregulated genes are indicated in red and blue, respectively. (**B**) Summary table of DEGs. LA-CB1 treatment led to 1502 upregulated and 2724 downregulated genes. (**C**) Heatmap of differentially expressed genes showing distinct clustering between LA-CB1-treated and control groups. (**D**) Gene Set Enrichment Analysis (GSEA) highlighting negatively enriched pathways, including EMT, E2F targets, and G2M checkpoint. (**E**) GSEA of additional pathways, including p53 signaling and Wnt/β-catenin pathways, which were downregulated following LA-CB1 treatment. (**F**) Gene Ontology (GO) enrichment analysis showing significant changes in pathways related to DNA damage response, cellular catabolic processes, and histone modification. (**G**) KEGG pathway enrichment analysis identifying affected pathways such as cell cycle regulation, Wnt signaling, and TGF-beta signaling. KEGG images cite this source: www.kegg.jp/kegg/kegg1.html.

To uncover the molecular mechanisms through which LA-CB1 exerts its anti-tumor effects, we performed transcriptomic analysis comparing LA-CB1-treated and DMSO-treated MDA-MB-231 cells. The volcano plot revealed that LA-CB1 treatment led to significant changes in gene expression, with 1502 genes upregulated and 2724 genes downregulated compared to the control group (Fig. 4A-B). Notably, key genes associated with tumor progression, metastasis, and cell cycle regulation were significantly affected. A heatmap of the differentially expressed genes (DEGs) confirmed clear separation between the LA-CB1-treated and DMSO-treated groups, indicating substantial transcriptional reprogramming induced by LA-CB1 (Fig. 4C). This transcriptional reprogramming likely contributes to the suppression of several malignancy-associated phenotypes such as cell proliferation, migration, and invasion. Gene Set Enrichment Analysis (GSEA) was conducted to provide further insights into the biological pathways affected by LA-CB1. The analysis revealed that LA-CB1 treatment significantly suppressed pathways related to EMT, suggesting that LA-CB1 disrupts the processes essential for cancer metastasis (Fig. 4D-E). Additionally, pathways associated with cell cycle regulation, including E2F targets and the G2M checkpoint, were negatively enriched following LA-CB1 treatment, indicating that LA-CB1 may inhibit MDA-MB-231 cell proliferation by arresting the cell cycle at critical checkpoints. This observation aligns with the previously noted G1 phase arrest in cell cycle studies. Furthermore, p53 signaling and Wnt/β-catenin pathways, both of which are involved in cell survival and oncogenic transformation, were downregulated in response to LA-CB1, further supporting the compound’s role in inducing apoptosis and inhibiting proliferative signaling in cancer cells. Gene Ontology (GO) enrichment analysis highlighted that LA-CB1 significantly impacted several key biological processes, including DNA damage response, cellular catabolic processes, and histone modification (Fig. 4F). The upregulation of genes involved in the DNA damage response suggests that LA-CB1 may enhance the susceptibility of cancer cells to DNA damage, potentially leading to apoptosis through the accumulation of unrepaired DNA lesions. Additionally, LA-CB1 altered pathways involved in histone modification, implying that the compound may exert epigenetic regulation, which could alter the expression of genes crucial for tumor cell survival and proliferation. Finally, KEGG pathway enrichment analysis provided further evidence that LA-CB1 affects critical oncogenic pathways. Cell cycle regulation, Wnt signaling, and TGF-beta signaling pathways were significantly impacted by LA-CB1, all of which play essential roles in driving tumorigenesis, promoting uncontrolled cell division, and metastasis (Fig. 4G). By downregulating these pathways, LA-CB1 likely inhibits the proliferative and metastatic potential of MDA-MB-231 cells. Notably, cellular senescence pathways were also enriched in response to LA-CB1, suggesting that the compound may promote tumor cell senescence, thereby reducing tumor growth and aggressiveness. These transcriptomic findings highlight that LA-CB1 exerts its anti-tumor effects by targeting multiple pathways critical for tumor malignancy, including EMT, cell cycle progression, and DNA damage response. By modulating these processes, LA-CB1 demonstrates the potential to suppress tumor proliferation, migration, invasion, and metastasis, offering valuable insights into its molecular mechanisms of action in cancer therapy.

### LA-CB1 induces cell cycle arrest and promotes apoptosis in breast Cancer cells

**Fig. 5:**
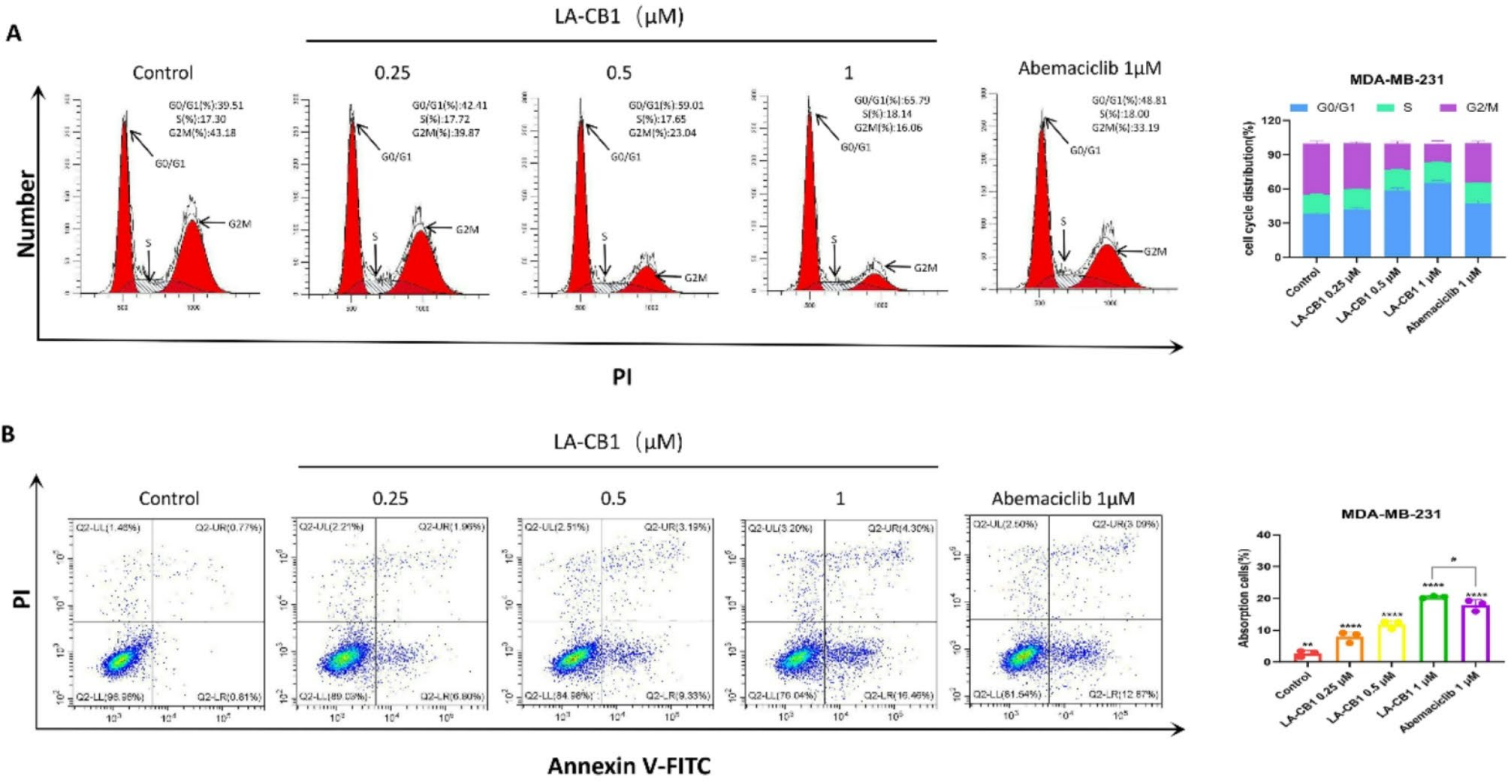
LA-CB1 Induces G0/G1 Cell Cycle Arrest and Apoptosis in MDA-MB-231 and MCF-7 Cells. (**A**) Cell cycle analysis of MDA-MB-231 cells treated with LA-CB1 (0.25, 0.5, 1 µM) for 48 h. Cells were stained with PI and analyzed by flow cytometry. LA-CB1 treatment induced G0/G1 phase arrest in a dose-dependent manner. (**B**) Annexin V-FITC/PI staining of MDA-MB-231 cells treated with LA-CB1 (0.25, 0.5, 1 µM) for 48 h. Representative flow cytometry plots show Annexin V and PI staining, indicating early and late apoptotic cells.

To dissect the underlying mechanisms responsible for LA-CB1’s potent anti-proliferative effects, detailed analyses of cell cycle regulation and apoptosis induction were conducted in MDA-MB-231 cells. These studies aimed to determine whether LA-CB1 exerts its anti-cancer activity by disrupting cell cycle progression and/or inducing programmed cell death. Flow cytometry was used to analyze the effects of LA-CB1 on the cell cycle distribution of cancer cells. MDA-MB-231 cells were treated with LA-CB1 at increasing concentrations (0.25, 0.5, and 1 µM) alongside Abemaciclib (1 µM) as a comparative control. In MDA-MB-231 cells, treatment with 1 µM LA-CB1 caused the G0/G1 phase population to increase to 65.79%, compared to 39.51% in the control group (*p* < 0.001) (Fig. 5A). This G0/G1 arrest was accompanied by a corresponding reduction in the G2/M phase population, suggesting that LA-CB1 effectively halts cell cycle progression at this critical checkpoint, thereby impairing the proliferative capacity of the MDA-MB-231 cells. In addition to the cell cycle arrest, the pro-apoptotic effects of LA-CB1 were examined using Annexin V-FITC/PI staining followed by flow cytometric analysis. MDA-MB-231 cells were exposed to the same concentrations of LA-CB1 as in the cell cycle study, with Abemaciclib serving as a control. The data revealed that LA-CB1 induced a significant, dose-dependent increase in apoptotic cell death in both cell lines. In MDA-MB-231 cells, treatment with 1 µM LA-CB1 induced apoptosis in 16.46% of cells, compared to 0.81% in the control group (*p* < 0.001) (Fig. 5B). These findings underscore the dual action of LA-CB1 in inhibiting cancer cell proliferation by inducing G0/G1 cell cycle arrest and promoting apoptosis, thereby contributing to its potent anti-cancer activity.

### LA-CB1 modulates cell cycle and apoptotic pathways in MDA-MB-231 and MCF-7 cells

**Fig. 6:**
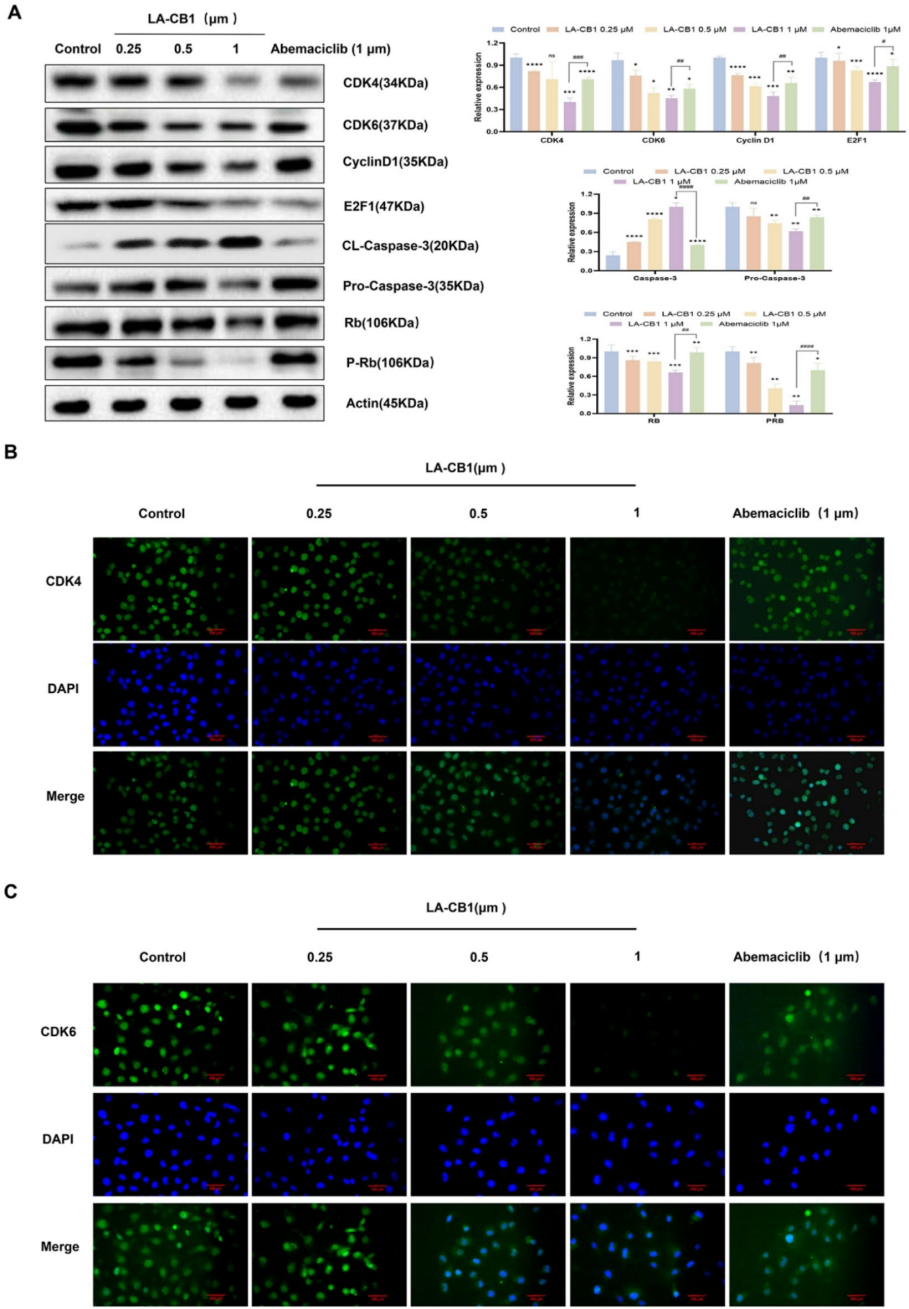
LA-CB1 Downregulates CDK4/6 and Cyclin D1, and Promotes Caspase-Dependent Apoptosis in MDA-MB-231 Cells. (**A**) Western blot analysis of CDK4, CDK6, Cyclin D1, E2F1, Rb, and cleaved caspase-3 in MDA-MB-231 cells treated with LA-CB1 (0.25, 0.5, 1 µM) for 48 h. LA-CB1 treatment resulted in dose-dependent downregulation of cell cycle regulators and activation of apoptotic pathways. (**B, C**) Immunofluorescence analysis of CDK4 (B) and CDK6 (C) in MDA-MB-231 cells treated with LA-CB1. Scale bar, 100 μm.

To further elucidate the molecular mechanisms underlying the effects of LA-CB1 on cell proliferation and survival, we performed Western blot and immunofluorescence assays to evaluate the expression of key proteins involved in cell cycle regulation and apoptosis. Western blot analysis showed that LA-CB1 treatment led to a dose-dependent downregulation of CDK4, CDK6, Cyclin D1, and E2F1, which are critical regulators of the G1/S transition in the cell cycle^4^ (Fig. 6A, ). At 1 µM, LA-CB1 significantly reduced the levels of CDK4 and CDK6 by more than 50%, suggesting that LA-CB1 exerts its effects by causing G1 phase arrest. In comparison, Abemaciclib at 1 µM similarly reduced the levels of these proteins, indicating that LA-CB1 may share a CDK4/6 inhibitory mechanism with Abemaciclib. Furthermore, phosphorylated Rb (P-Rb), the active form that promotes cell cycle progression, was significantly decreased following LA-CB1 treatment, further confirming the induction of G1 phase arrest (Fig. 6A, Supplementary Fig. 6A). In addition to its effects on cell cycle regulators, LA-CB1 modulated apoptosis-related proteins in both cell lines. Western blot analysis revealed a dose-dependent increase in cleaved caspase-3 and a decrease in pro-caspase-3, indicating the activation of the caspase-dependent apoptosis pathway (Fig. 6A, Supplementary Fig. 6A). At 1 µM, LA-CB1 induced significant caspase-3 cleavage, comparable to the effects observed with Abemaciclib, suggesting that LA-CB1 not only inhibits cell proliferation but also actively promotes apoptotic cell death. The immunofluorescence analysis confirmed the findings from the Western blot studies. In both MDA-MB-231 and MCF-7 cells, LA-CB1 treatment resulted in a marked reduction in the expression of CDK4 and CDK6 in a dose-dependent manner (Fig. 6B-C, Supplementary Fig. 6B-C). At 1 µM, the fluorescence intensity of CDK4 and CDK6 was significantly lower compared to the control and lower doses of LA-CB1, further corroborating the results from the Western blot analysis and supporting the hypothesis that LA-CB1 induces G1 phase arrest by targeting CDK4/6.Together, these data indicate that LA-CB1 exerts its anti-tumor effects through a dual mechanism: (1) by inhibiting cell cycle progression via the downregulation of CDK4/6 and Cyclin D1, and (2) by promoting apoptosis through the activation of caspase-3. These findings suggest that LA-CB1 has the potential to target key regulatory pathways involved in tumor growth and survival in both MDA-MB-231 and MCF-7 breast cancer cell lines.

### LA-CB1 promotes CDK4/6 degradation via the Ubiquitin-Proteasome pathway

**Fig. 7:**
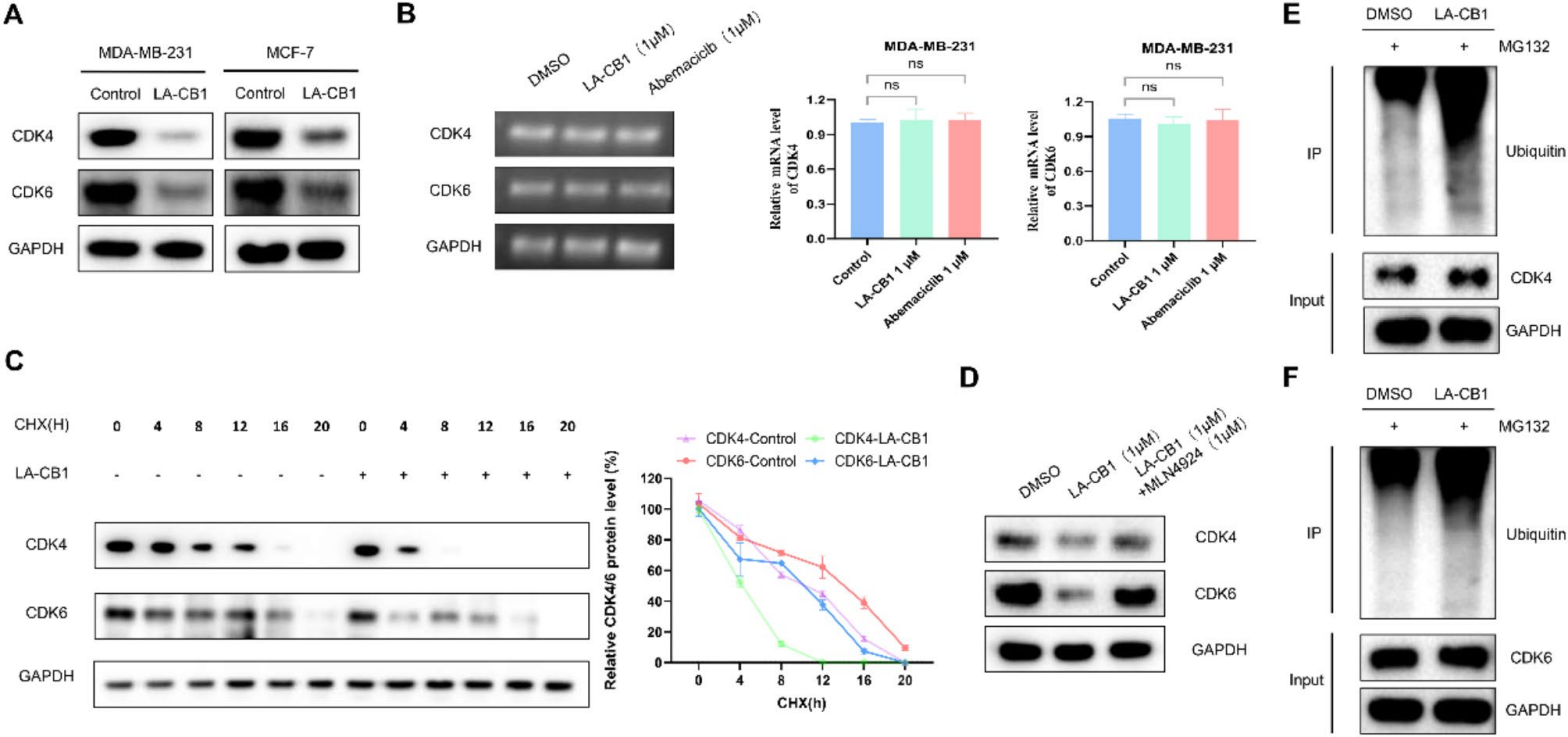
LA-CB1 Promotes CDK4/6 Degradation via the Ubiquitin-Proteasome Pathway. (**A**) Western blot analysis of CDK4 and CDK6 in MDA-MB-231 and MCF-7 cells treated with LA-CB1. LA-CB1 significantly reduced the levels of CDK4 and CDK6 proteins. (**B**) RT-PCR analysis of CDK4 and CDK6 mRNA levels in MDA-MB-231 cells treated with LA-CB1. No significant changes in mRNA levels were observed, indicating that the downregulation of CDK4/6 occurs at the post-transcriptional level. (**C**) Cycloheximide chase assay showing the degradation kinetics of CDK4 and CDK6 in the presence of LA-CB1. LA-CB1 promoted the degradation of CDK4/6 in a time-dependent manner. (**D**) MDA-MB-231 cells were treated with 1 µM LA-CB1 for 24 h and 1 µM MLN4924 was added 4 h prior to harvesting the cells.After harvesting, cell lysates were prepared and protein was detected using the indicated antibodies. (**E, F**) Immunoprecipitation followed by Western blot analysis of CDK4 (D) and CDK6 (E) ubiquitination in MDA-MB-231 cells treated with LA-CB1 and MG132. Increased ubiquitination of both CDK4 and CDK6 was observed following LA-CB1 treatment, indicating that LA-CB1 promotes ubiquitin-proteasome-mediated degradation of these proteins.

To further investigate the mechanism by which LA-CB1 downregulates CDK4/6 expression, we assessed the effects of LA-CB1 on CDK4/6 protein stability and degradation pathways. Western blot analysis revealed that LA-CB1 significantly decreased the protein levels of CDK4 and CDK6 in both cell lines compared to the control (Fig. 7A). However, RT-PCR analysis showed that LA-CB1 treatment did not significantly affect the mRNA levels of CDK4 and CDK6, suggesting that the downregulation of these proteins occurs at the post-transcriptional level (Fig. 7B). To determine whether LA-CB1 affects the stability of CDK4/6, cells were treated with cycloheximide (CHX), a protein synthesis inhibitor, and the degradation rate of CDK4/6 was measured over time. In the presence of LA-CB1, the degradation rate of both CDK4 and CDK6 was significantly increased, as indicated by the faster decline in protein levels compared to the control group (Fig. 7C). This suggests that LA-CB1 promotes the proteasomal degradation of CDK4/6. MLN4924 is a small molecule inhibitor targeting NEDD8 activating enzyme (NAE). By inhibiting NAE, MLN4924 blocks the activation of NEDD8, which in turn inhibits neddylation of Cullin-RING ubiquitin ligases (CRLs) (NEDD8 conjugation). Next, when we added both LA-CB1 and MLN4924 the results showed that MLN4924 was able to have a reversal effect on the degradation of CDK4/6 by LA-CB1 (Fig. 7D), which suggests that the ubiquitinated degradation of CDK4/6 by LA-CB1 may be a Cullin-RING ubiquitin ligase. Next, we explored whether LA-CB1 induces CDK4/6 degradation through the ubiquitin-proteasome pathway. Cells were treated with the proteasome inhibitor MG132, in the presence or absence of LA-CB1. Immunoprecipitation followed by Western blot analysis demonstrated increased ubiquitination of both CDK4 and CDK6 in LA-CB1-treated cells compared to controls (Fig. 7E-F). These results indicate that LA-CB1 downregulates CDK4/6 by promoting their degradation via the ubiquitin-proteasome pathway, rather than through transcriptional repression. This post-translational regulation of CDK4/6 is likely responsible for the cell cycle arrest observed in G1 phase, further supporting the therapeutic potential of LA-CB1 as a cancer treatment targeting CDK4/6-mediated proliferation.

### LA-CB1 inhibits tumor growth in an orthotopic 4T1 breast Cancer model

**Fig. 8:**
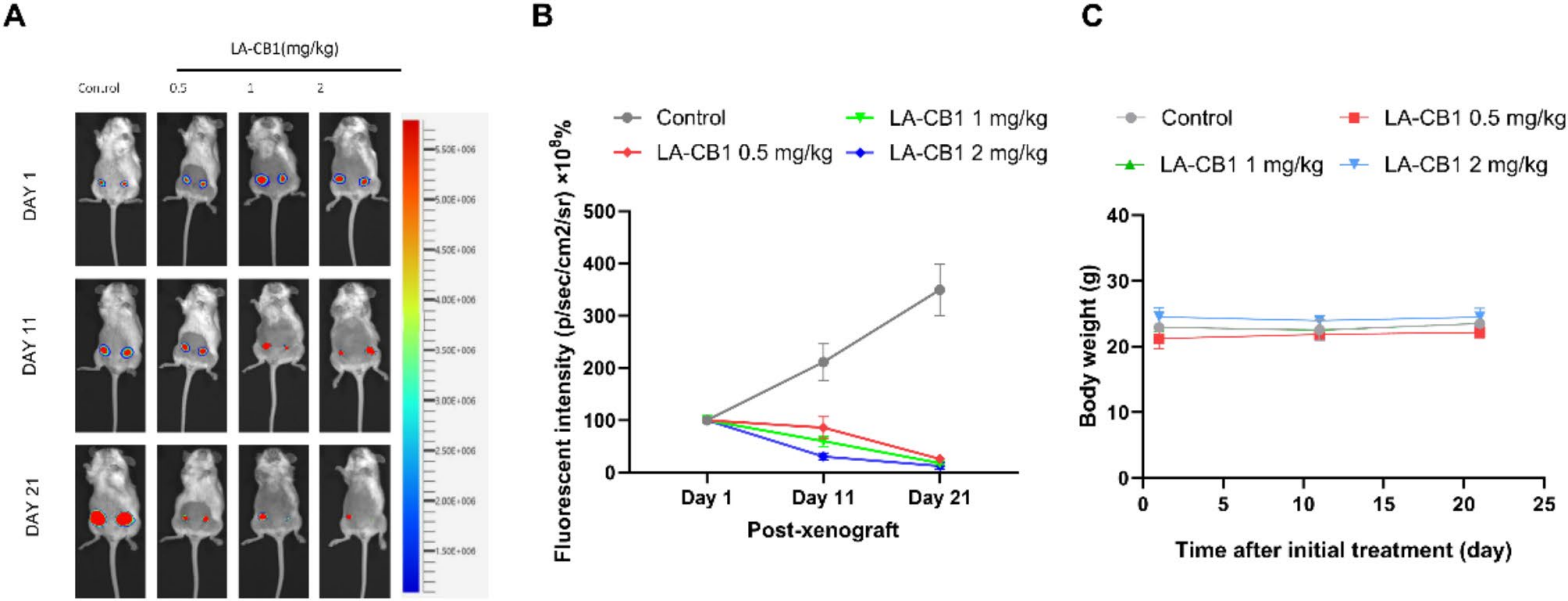
LA-CB1 Suppresses Tumor Growth in an Orthotopic Breast Cancer Model in Mice. (**A**) Bioluminescence imaging of 4T1-LUC tumors in mice treated with varying doses of LA-CB1 (0.5 mg/kg, 1 mg/kg, 2 mg/kg) or vehicle control at Day 1, Day 11, and Day 21 post-xenograft. (**B**) Quantification of bioluminescent signal intensity from tumors in each group. LA-CB1 significantly reduced tumor bioluminescence in a dose-dependent manner. *p* < 0.001 for the 2 mg/kg LA-CB1 group compared to the control group. Data are represented as mean ± SD (*n* = 6). (**C**) Body weight measurements during treatment.

The in vivo anti-tumor efficacy of LA-CB1 was evaluated using an orthotopic breast cancer model with 4T1-LUC cells in ICR mice. 4T1-LUC cells, which stably express luciferase, were injected into the mammary fat pad of the mice to mimic the tumor microenvironment. Following the establishment of the tumors, mice were treated with varying doses of LA-CB1 (0.5 mg/kg, 1 mg/kg, and 2 mg/kg) or vehicle control, and bioluminescence imaging was used to monitor tumor growth at Day 1, Day 11, and Day 21 post-xenograft. The bioluminescence images demonstrated a clear dose-dependent inhibition of tumor growth in the LA-CB1-treated groups compared to the control group (Fig. 8A). At Day 1, bioluminescence signals in the treatment groups were similar to those in the control group, indicating that initial tumor sizes were comparable across all groups. However, by Day 11, there was a marked reduction in tumor bioluminescence in the LA-CB1-treated groups, with the 2 mg/kg LA-CB1 group showing the most significant reduction in tumor burden. By Day 21, tumors in the control group exhibited a substantial increase in bioluminescence signal, indicating aggressive tumor progression, whereas all LA-CB1-treated groups showed significantly reduced tumor bioluminescence, with the 2 mg/kg LA-CB1 group displaying the lowest signal, reflecting the greatest inhibition of tumor growth. Quantitative analysis of the bioluminescent signal confirmed that LA-CB1 treatment significantly suppressed tumor growth in a dose-dependent manner (Fig. 8B). No severe weight loss was observed during the treatment period (Fig. 8C). At Day 21, the bioluminescent intensity in the 2 mg/kg LA-CB1 group was reduced by more than 80% compared to the control group (*p* < 0.001). Similarly, the 1 mg/kg and 0.5 mg/kg LA-CB1 groups also showed significant tumor inhibition, with bioluminescence reductions of approximately 60% and 40%, respectively, compared to the control (*p* < 0.01). These results demonstrate that LA-CB1 exhibits potent in vivo anti-tumor activity in this orthotopic model of breast cancer. These findings highlight the potential of LA-CB1 as an effective therapeutic agent for inhibiting tumor growth in aggressive breast cancer models. The ability of LA-CB1 to significantly reduce tumor burden in a dose-dependent manner, particularly at higher doses, underscores its potential application as a treatment for advanced-stage cancers. Further in vivo studies focusing on survival, metastasis prevention, and combination therapies could provide additional insights into the therapeutic potential of LA-CB1 in clinical settings.

## Discussion

**Fig. 9:**
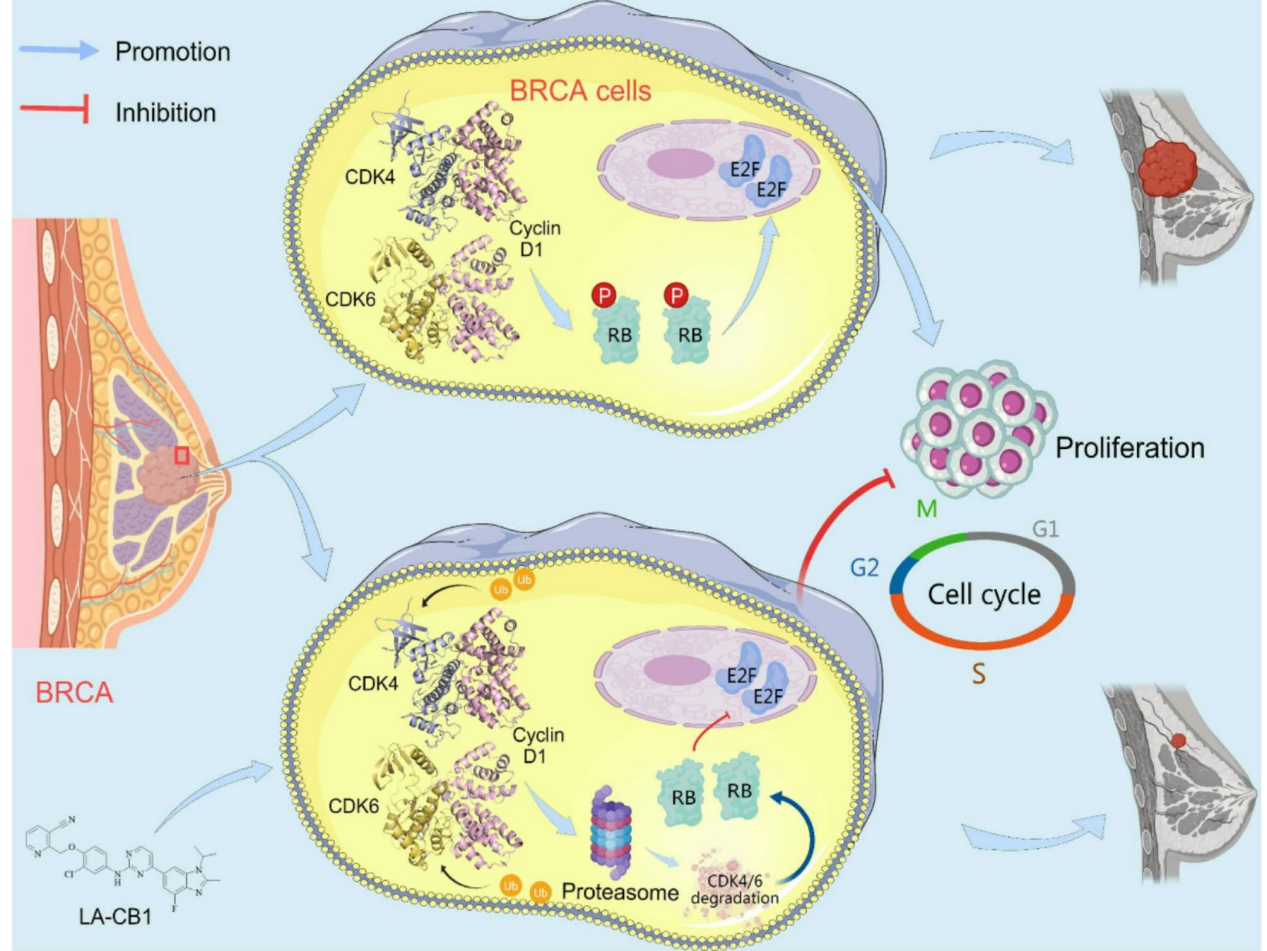
Working model. This working model, based on our experimental findings, proposes the mechanism by which LA-CB1 exerts its anti-tumor activity in breast cancer (BRCA) cells through the targeted degradation of CDK4/6. In untreated BRCA cells (top panel), CDK4/6 forms complexes with Cyclin D1, leading to the phosphorylation of the retinoblastoma protein (Rb). Phosphorylated Rb releases E2F transcription factors, which activate the transcription of genes involved in DNA replication and cell cycle progression. This process allows the cells to transition from the G1 phase to the S phase, driving cell proliferation and tumor growth. In the presence of LA-CB1 (bottom panel), this pathway is disrupted. LA-CB1 promotes the ubiquitination and subsequent proteasomal degradation of CDK4/6, preventing the formation of the CDK4/6-Cyclin D1 complex. This inhibition blocks Rb phosphorylation, thereby sequestering E2F in its inactive state, bound to unphosphorylated Rb. As a result, cell cycle progression is halted at the G1 phase, preventing the transition to the S phase and inhibiting cancer cell proliferation. This arrest in the G1/S transition effectively suppresses tumor growth. This model underscores the potential of LA-CB1 as a therapeutic agent that not only inhibits CDK4/6 activity but also promotes their degradation, offering a novel approach to disrupting cell cycle progression in breast cancer, including triple-negative breast cancer where targeted therapies remain limited. This mechanistic insight provides a rationale for the development of LA-CB1 as a potential anti-cancer agent capable of addressing CDK4/6-driven malignancies.

In this study, we identified LA-CB1 as a potent and novel inhibitor of cancer cell proliferation, with a unique mechanism of action involving the ubiquitin-proteasome-mediated degradation of CDK4/6 (Fig. 9). Our findings demonstrate that LA-CB1 exerts robust anti-proliferative effects across a wide spectrum of cancer cell lines. Compared to established CDK inhibitors such as Abemaciclib, Ribociclib, and Palbociclib, LA-CB1 exhibited significantly lower IC50 values, underscoring its superior potency. Importantly, LA-CB1 was shown to induce G0/G1 cell cycle arrest, promote apoptosis, and inhibit key processes involved in tumor progression, including cell migration, invasion, and angiogenesis. The ability of LA-CB1 to degrade CDK4/6, rather than merely inhibit its catalytic activity, offers a mechanistic advantage, potentially enabling more sustained suppression of cell cycle progression and reducing the likelihood of resistance—an issue frequently encountered with conventional CDK inhibitors^5,29^.

Molecular docking studies further suggested that LA-CB1’s enhanced binding affinity to CDK4/6, facilitated by an additional hydrogen bond interaction via its cyano group, may be a critical factor contributing to its superior efficacy. In vivo studies using an orthotopic 4T1-LUC breast cancer xenograft model confirmed LA-CB1’s therapeutic potential, as treatment with LA-CB1 significantly reduced tumor burden. Bioluminescence imaging indicated dose-dependent inhibition of tumor growth, which is particularly promising for the treatment of aggressive malignancies such as triple-negative breast cancer. Furthermore, LA-CB1’s ability to inhibit angiogenesis and modulate EMT markers, suggests that this compound may possess broader anti-metastatic properties, positioning it as a strong candidate for preventing cancer dissemination.

A key advantage of our approach lies in circumventing the limitations of PROTAC-based strategies, which have garnered attention for their ability to induce protein degradation but face several practical challenges. The large and structurally complex nature of PROTAC molecules often results in poor cell permeability and suboptimal pharmacokinetics^30,31^. Furthermore, PROTACs depend on a dual-binding mechanism, requiring simultaneous engagement with both the target protein and an E3 ligase. This limits their versatility and efficacy, particularly in cases where the expression of the target protein or E3 ligase is inadequate^32,33^. In contrast, our non-PROTAC approach employs a simplified chemical structure and a distinct mechanism, improving bioavailability, intracellular distribution, and target specificity. This enhanced flexibility in targeting CDK4/6 through degradation, rather than inhibition, allows for a broader range of therapeutic applications across various cancer types.

Another key advantage of LA-CB1 is its reduced potential for off-target effects and toxicity. PROTACs, due to their dual-binding nature, may inadvertently degrade non-specific proteins, leading to undesirable side effects^34,35^. Our non-PROTAC approach allows for precise molecular targeting, minimizing off-target activity and potentially improving the therapeutic index of LA-CB1.

Despite these promising results, several limitations of the current study must be addressed. First, while LA-CB1 demonstrated robust efficacy both in vitro and in vivo, its pharmacokinetic properties and toxicity profile have yet to be fully characterized. Although CDK inhibitors generally exhibit favorable safety profiles, the novel mechanism of CDK4/6 degradation by LA-CB1 could potentially lead to unforeseen side effects in normal tissues where CDK4/6 plays essential roles in homeostasis. Comprehensive pharmacokinetic and toxicological evaluations will be essential to assess LA-CB1’s safety, particularly in the context of long-term treatments and in tissues dependent on CDK4/6 function.

Although the difference between Abemaciclib and LA-CB1 was statistically significant, the biological significance of this effect is unclear. Statistical significance suggests that the observed difference is unlikely to be due to random error, but by Fig. 5 The statistically significant difference between Abemaciclib and LA-CB1 (*p* < 0.05), but with an effect size of 0.25 as measured by Cohen’s d suggests that, although the difference is statistically reliable, the practical or biological significance of the observed effect may be limited. It can be seen that the effect size of the difference may be small and may not be significant at the biological level. Further studies are necessary to assess whether these differences can be translated into clinically meaningful outcomes, especially in areas such as cell proliferation. This will require us to further validate whether these statistical differences are biologically relevant by increasing the sample size and diversifying the experimental models in the future.

Additionally, this study did not explore potential resistance mechanisms that could emerge following prolonged LA-CB1 treatment. While LA-CB1 effectively induces CDK4/6 degradation, tumors may develop compensatory pathways that bypass CDK4/6 inhibition, a phenomenon commonly observed with other targeted therapies^36^. Future research should investigate potential resistance mechanisms and evaluate combination therapies, such as pairing LA-CB1 with immune checkpoint inhibitors or standard chemotherapy, to enhance the durability of therapeutic responses. Expanding the study to other tumor types reliant on CDK4/6 signaling, such as melanoma, lung cancer, and colorectal cancer, will also be important to fully realize LA-CB1’s clinical potential.

Moreover, future studies should focus on optimizing the chemical structure of LA-CB1 through structure-activity relationship (SAR) analyses, aiming to further improve its pharmacokinetic properties, selectivity, and binding affinity. Such optimization may enhance its therapeutic index, minimize off-target effects, and broaden its clinical utility. Additionally, employing more clinically relevant models, such as patient-derived xenografts (PDX) or spontaneous metastasis models, will be crucial for assessing LA-CB1’s efficacy in more complex tumor microenvironments and determining its applicability across diverse cancer subtypes.

In conclusion, LA-CB1 represents a novel class of CDK4/6-targeting agents, distinguished by its ability to induce proteasomal degradation of these kinases. The compound exhibited potent anti-proliferative, anti-angiogenic, and anti-metastatic activities both in vitro and in vivo, positioning it as a highly promising candidate for the treatment of aggressive and metastatic cancers. While further preclinical studies are required to fully elucidate its safety and therapeutic potential, our findings provide compelling evidence supporting the continued development of LA-CB1 as a novel cancer therapeutic agent.

## Electronic supplementary material

Below is the link to the electronic supplementary material.

Supplementary Material 1

Supplementary Material 2

## Data Availability

The RNA-Seq dataset generated during the current study are available in the in the Gene Expression Omnibus repository, (the accession number: GSE276256), and the link of the repository, https://www.ncbi.nlm.nih.gov/geo/query/acc.cgi? acc=GSE276256.
