# Supplementary material for: Targeted degradation of CDK4/6 by LA-CB1 inhibits EMT and suppresses tumor growth in orthotopic breast cancer
Source: Sci Rep. 2025 Mar 4;15:7605. doi: 10.1038/s41598-025-92494-8 (PMC11880390; doi:10.1038/s41598-025-92494-8)

Figure 3I

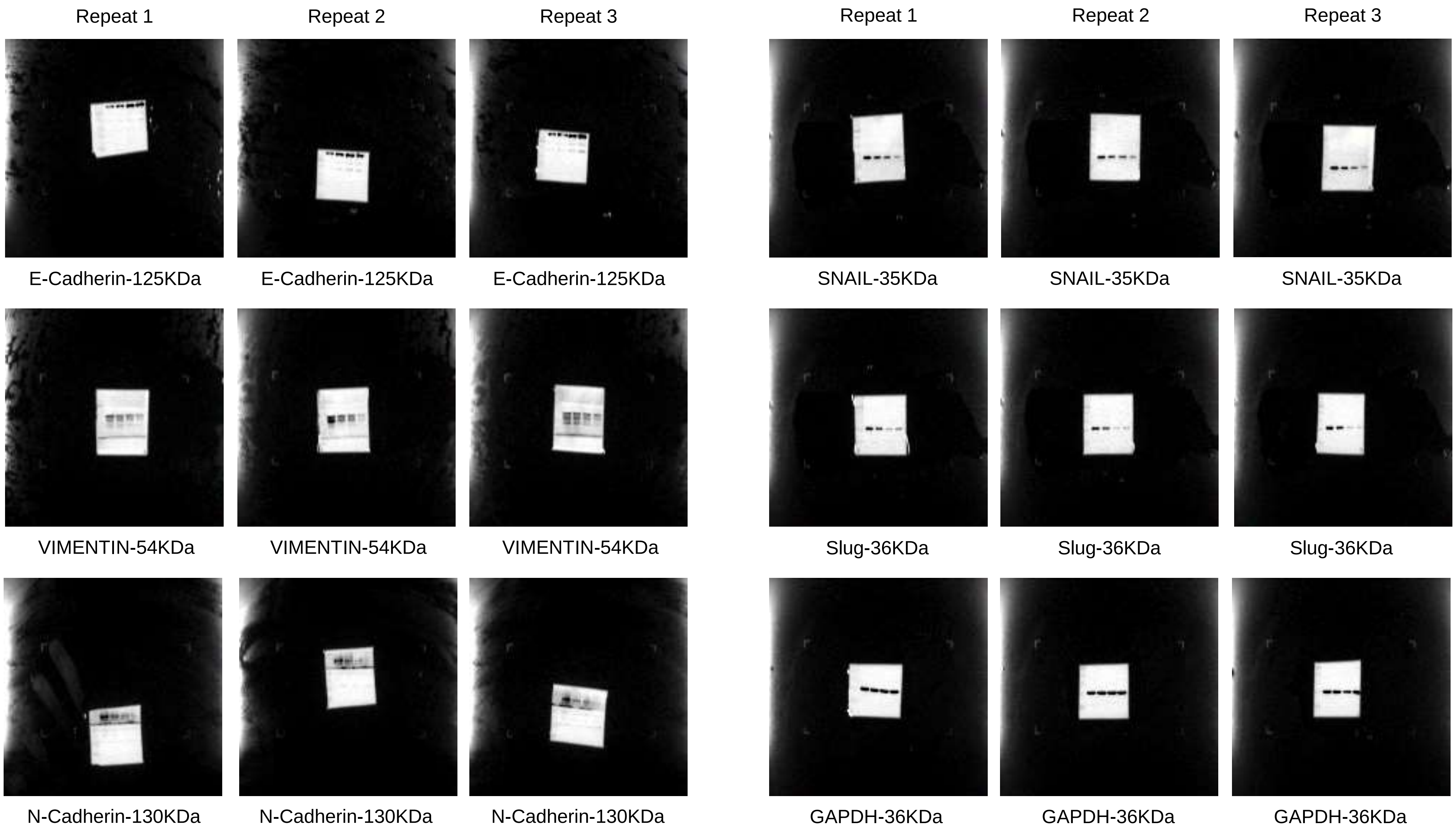
Repeat 1
Repeat 2
Repeat 3
Repeat 1
Repeat 2
Repeat 3
E-Cadherin-125KDa
E-Cadherin-125KDa
E-Cadherin-125KDa
SNAIL-35KDa
SNAIL-35KDa
SNAIL-35KDa
VIMENTIN-54KDa
VIMENTIN-54KDa
VIMENTIN-54KDa
Slug-36KDa
Slug-36KDa
Slug-36KDa
N-Cadherin-130KDa
N-Cadherin-130KDa
N-Cadherin-130KDa
GAPDH-36KDa
GAPDH-36KDa
GAPDH-36KDa

Figure 6A

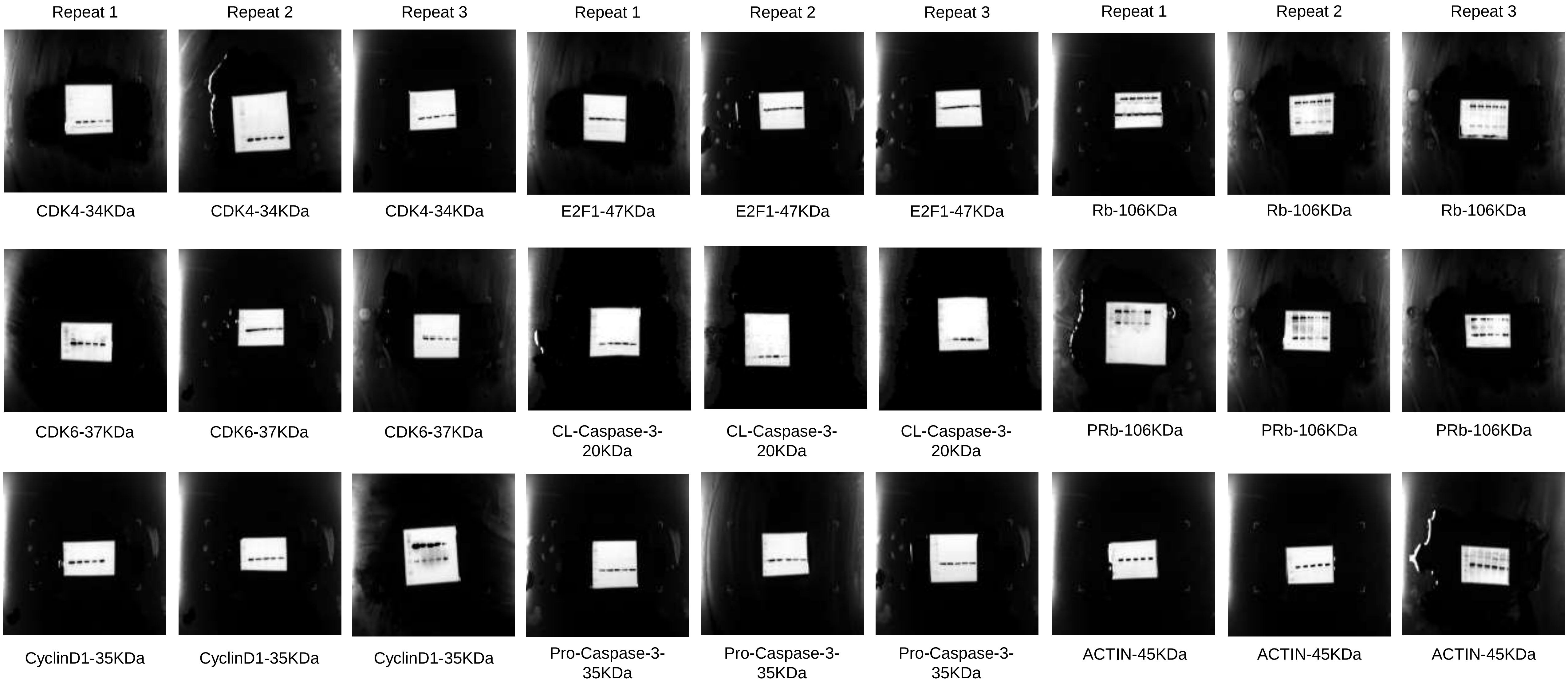
Repeat 1
Repeat 2
Repeat 3
Repeat 1
Repeat 2
Repeat 3
Repeat 1
Repeat 2
Repeat 3
CDK4-34KDa
CDK4-34KDa
CDK4-34KDa
E2F1-47KDa
E2F1-47KDa
E2F1-47KDa
Rb-106KDa
Rb-106KDa
Rb-106KDa
CDK6-37KDa
CDK6-37KDa
CDK6-37KDa
CL-Caspase-3-20KDa
CL-Caspase-3-20KDa
CL-Caspase-3-20KDa
PRb-106KDa
PRb-106KDa
PRb-106KDa
CyclinD1-35KDa
CyclinD1-35KDa
CyclinD1-35KDa
Pro-Caspase-3-35KDa
Pro-Caspase-3-35KDa
Pro-Caspase-3-35KDa
ACTIN-45KDa
ACTIN-45KDa
ACTIN-45KDa

Figure 7A

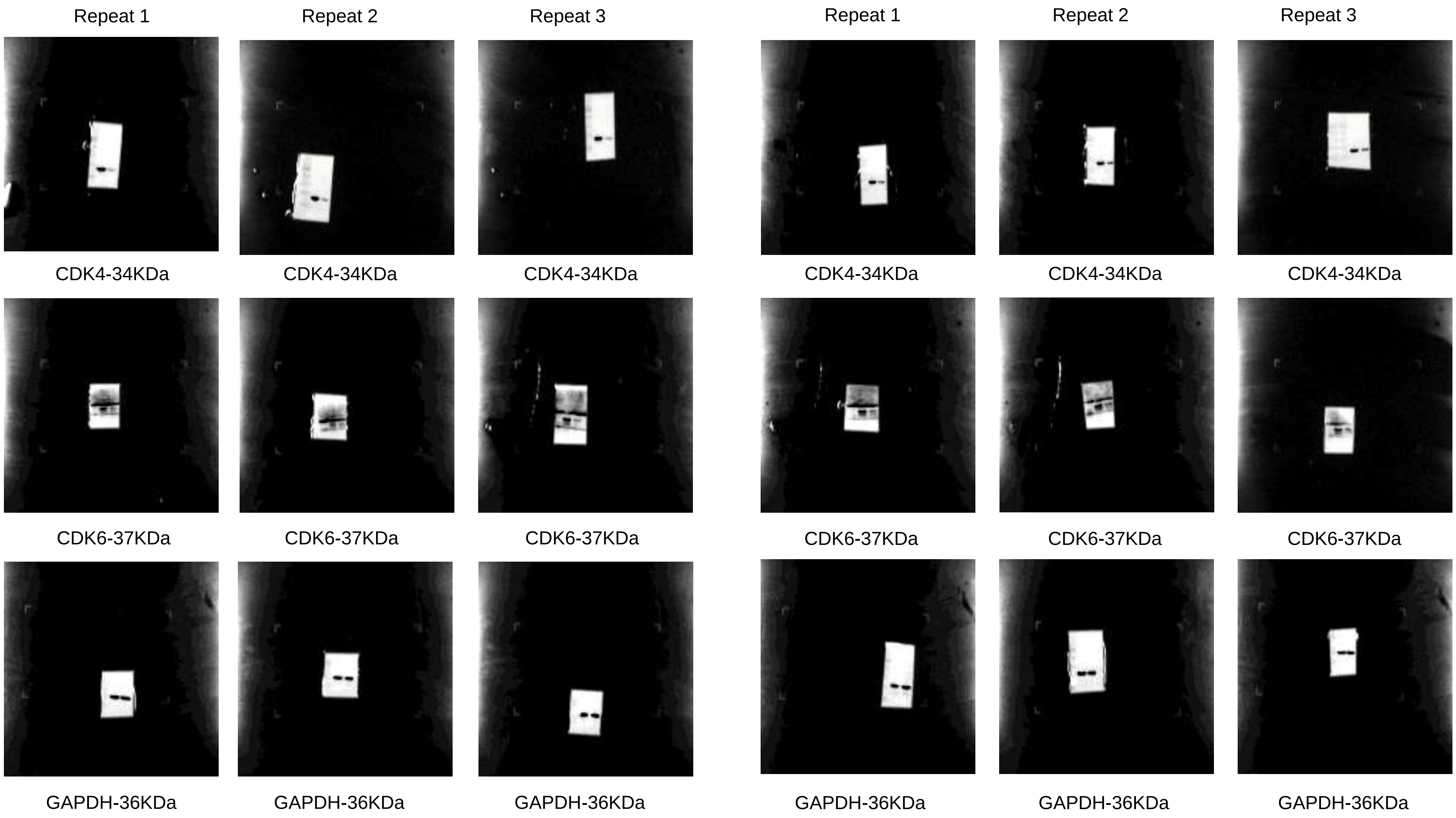
Repeat 1
Repeat 2
Repeat 3
Repeat 1
Repeat 2
Repeat 3
CDK4-34KDa
CDK4-34KDa
CDK4-34KDa
CDK4-34KDa
CDK4-34KDa
CDK4-34KDa
CDK6-37KDa
CDK6-37KDa
CDK6-37KDa
CDK6-37KDa
CDK6-37KDa
CDK6-37KDa
GAPDH-36KDa
GAPDH-36KDa
GAPDH-36KDa
GAPDH-36KDa
GAPDH-36KDa
GAPDH-36KDa

Figure 7B

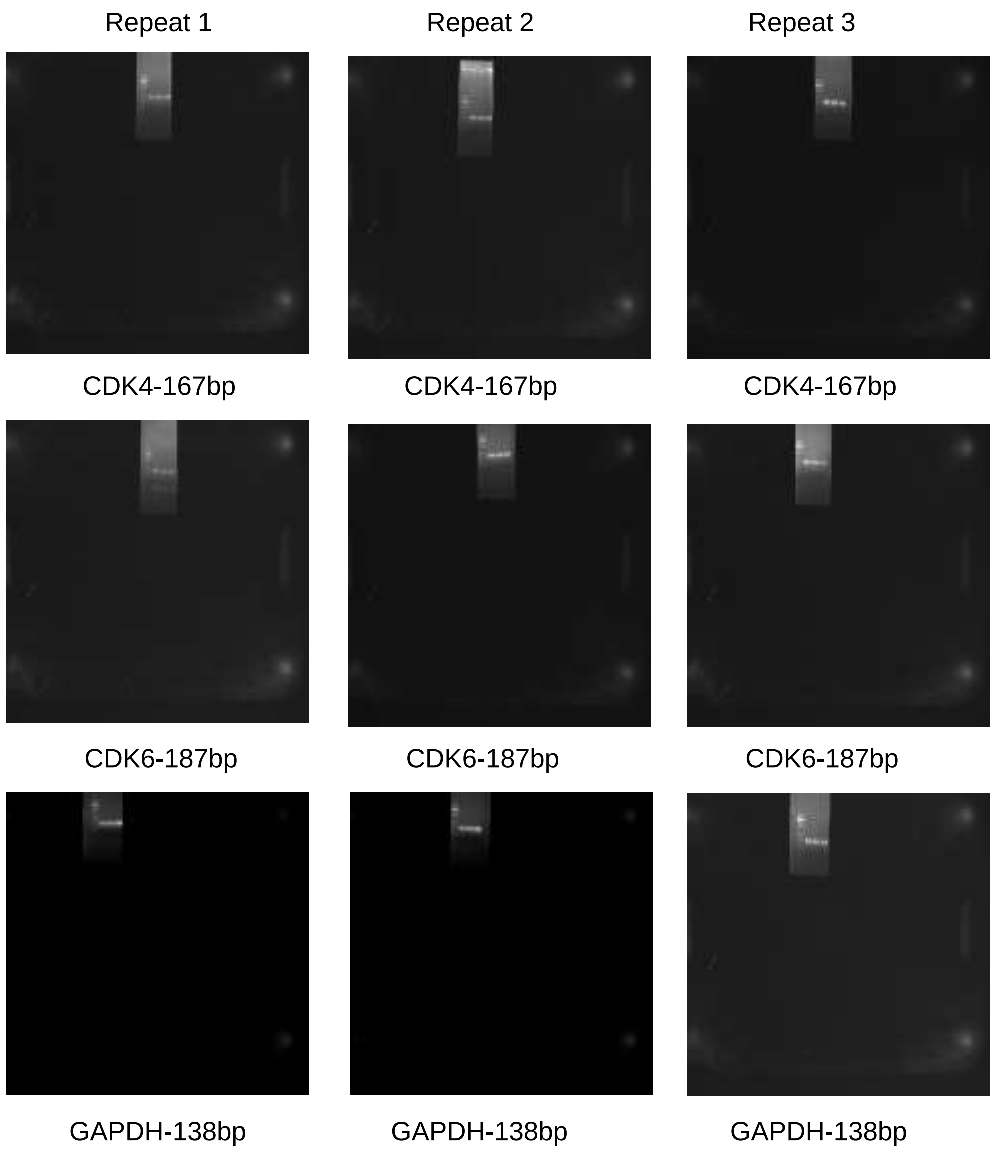
Repeat 1
Repeat 2
Repeat 3
CDK4-167bp
CDK4-167bp
CDK4-167bp
CDK6-187bp
CDK6-187bp
CDK6-187bp
GAPDH-138bp
GAPDH-138bp
GAPDH-138bp

Figure 7C

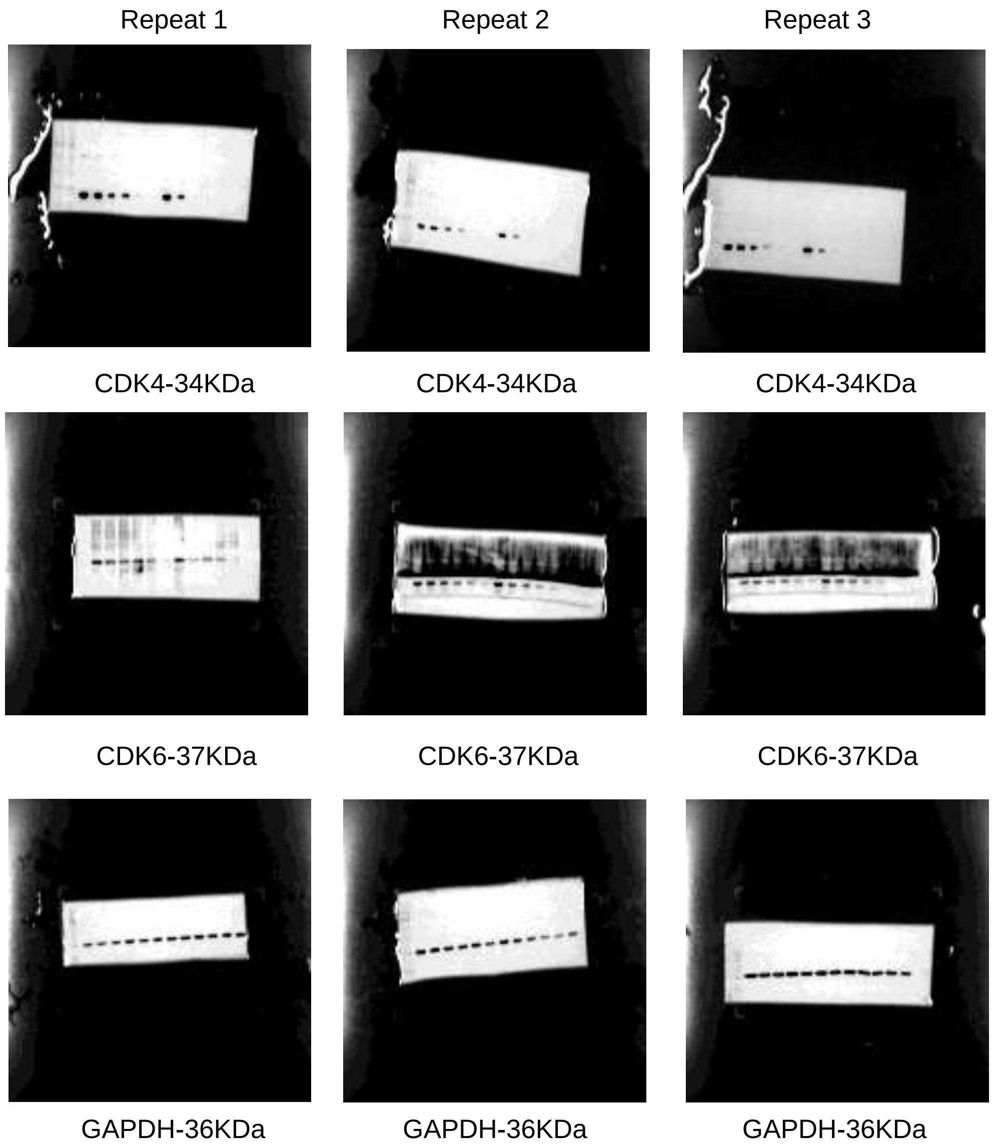
Repeat 1
Repeat 2
Repeat 3
CDK4-34KDa
CDK4-34KDa
CDK4-34KDa
CDK6-37KDa
CDK6-37KDa
CDK6-37KDa
GAPDH-36KDa
GAPDH-36KDa
GAPDH-36KDa

Figure 7D

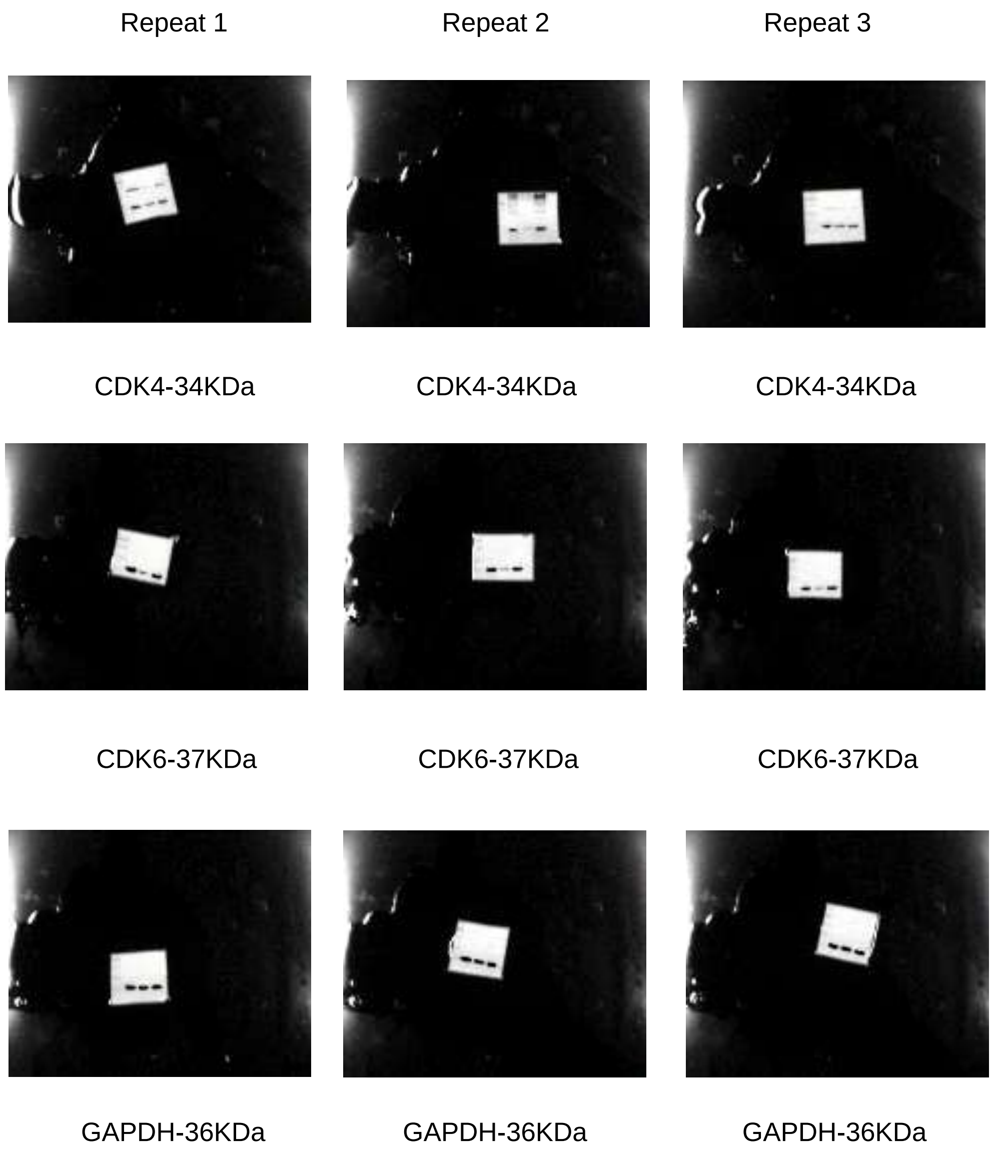
Repeat 1
Repeat 2
Repeat 3
CDK4-34KDa
CDK4-34KDa
CDK4-34KDa
CDK6-37KDa
CDK6-37KDa
CDK6-37KDa
GAPDH-36KDa
GAPDH-36KDa
GAPDH-36KDa

Figure 7E,F

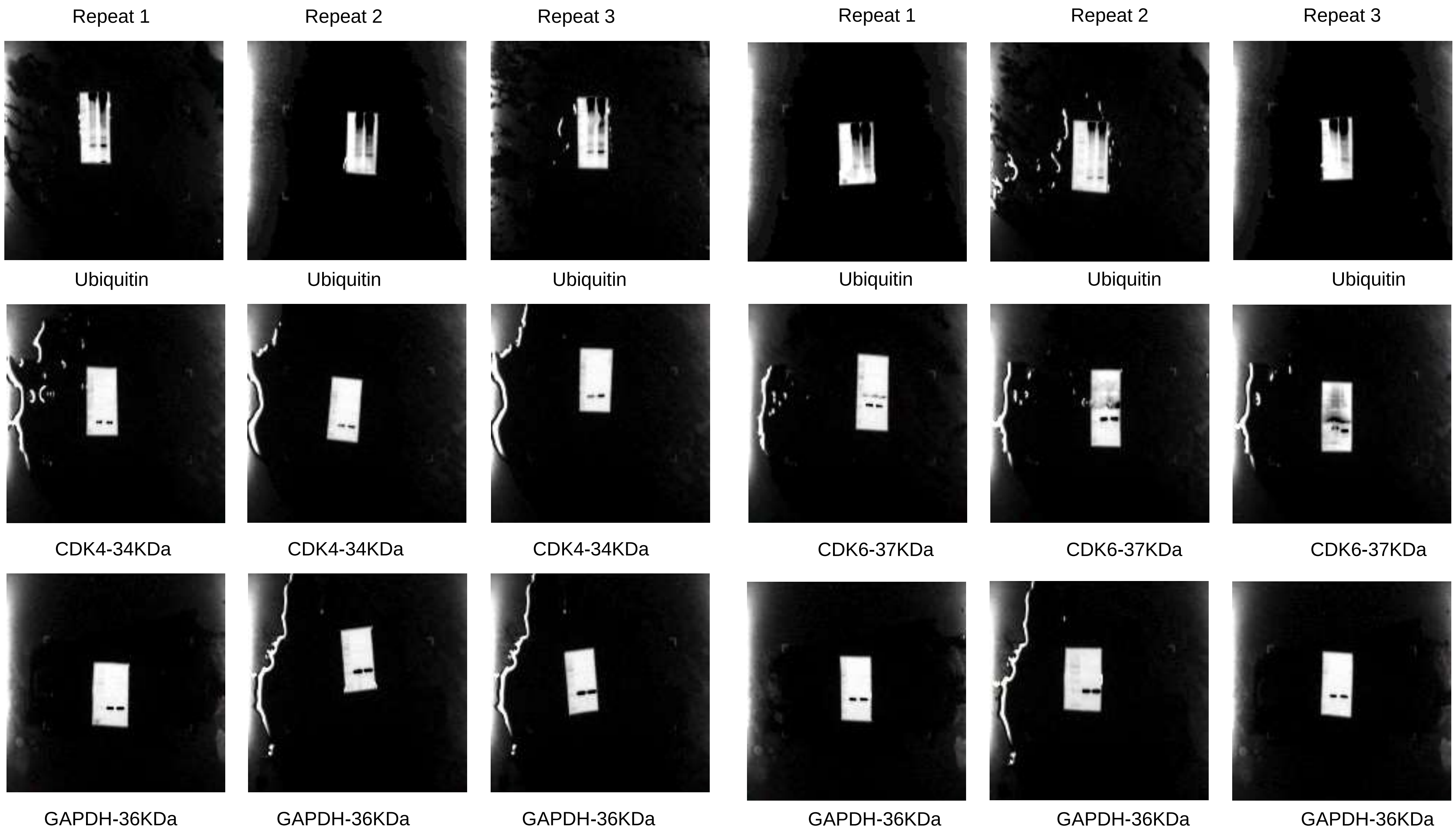
Repeat 1
Repeat 2
Repeat 3
Repeat 1
Repeat 2
Repeat 3
Ubiquitin
Ubiquitin
Ubiquitin
Ubiquitin
Ubiquitin
Ubiquitin
CDK4-34KDa
CDK4-34KDa
CDK4-34KDa
CDK6-37KDa
CDK6-37KDa
CDK6-37KDa
GAPDH-36KDa
GAPDH-36KDa
GAPDH-36KDa
GAPDH-36KDa
GAPDH-36KDa
GAPDH-36KDa

Supplementary Figure 3I

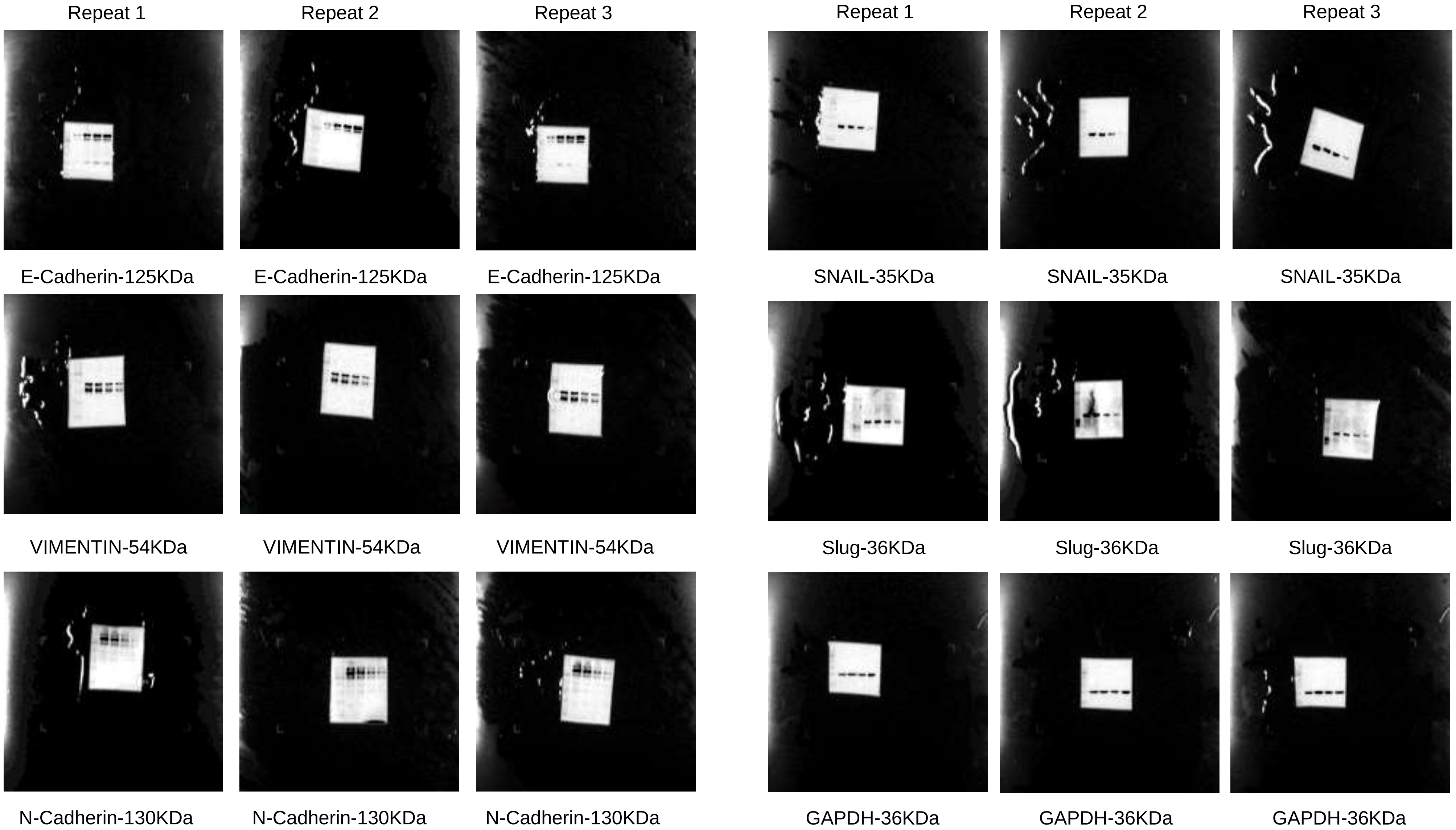

Supplementary Figure 4A

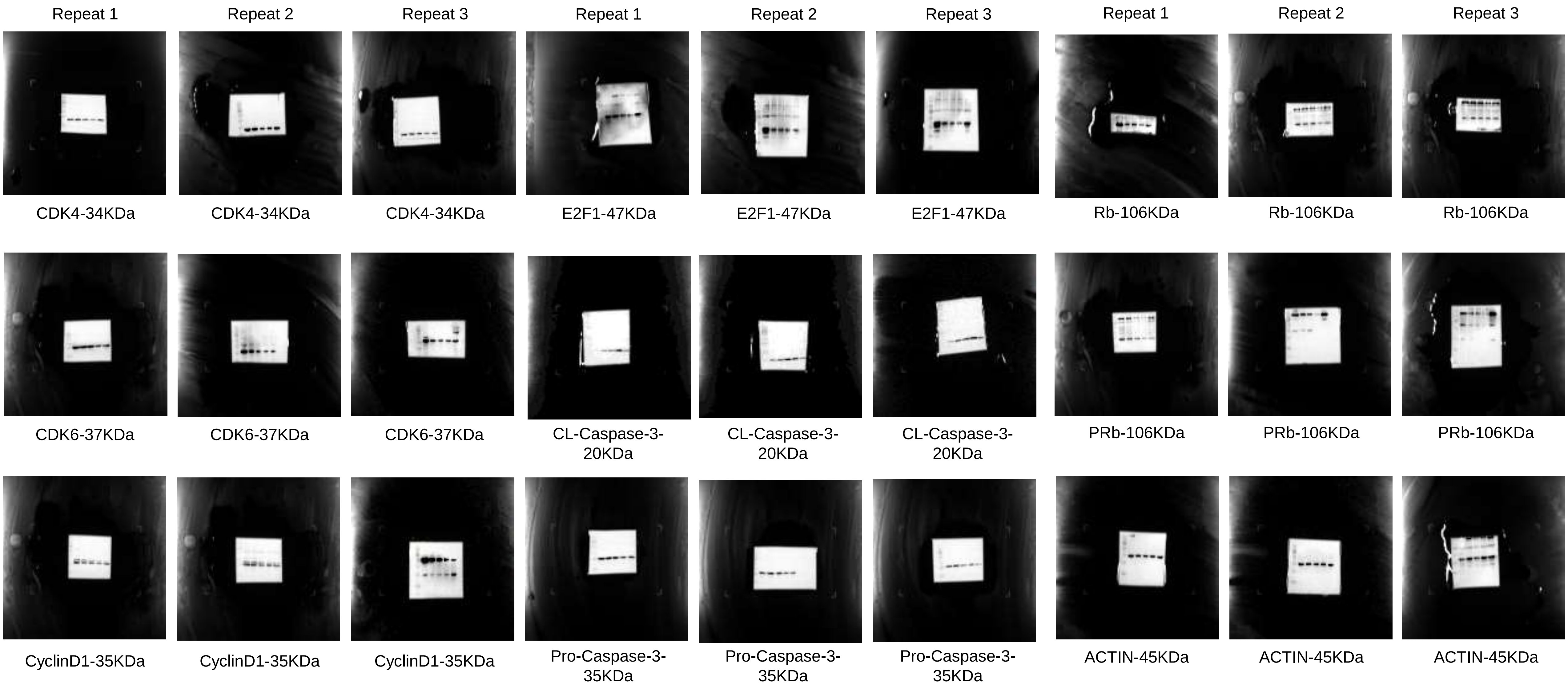

Supplement: Supplementary file 1 — Supplementary Material 1 [file 41598_2025_92494_MOESM1_ESM.pdf]
