# Supplementary material for: Targeted degradation of CDK4/6 by LA-CB1 inhibits EMT and suppresses tumor growth in orthotopic breast cancer
Source: Sci Rep. 2025 Mar 4;15:7605. doi: 10.1038/s41598-025-92494-8 (PMC11880390; doi:10.1038/s41598-025-92494-8)
Supplement: Supplementary file 2 — Supplementary Material 2 [file 41598_2025_92494_MOESM2_ESM.docx]

**Targeted Degradation of CDK4/6 by LA-CB1 Inhibits EMT and Suppresses Tumor Growth in Orthotopic Breast Cancer**

Jingliang He^1#^, Shunfang Liu^2#^, Siyi Zhang^1#^, Qi Gao^1#^, Lan Zhu ^3^, Ningyang Xu^3^, Zhongke Hu^1^, Xingyu Zhang^1^, Shaojie Ma^1^, Xiujun Wang^1^, Bin Liu^1*^, Wei Liu^3*^

1. Jiangsu Key Laboratory of Marine Pharmaceutical Compound Screening, College of Pharmacy, Jiangsu Ocean University, Lianyungang, 222005, China.
2. Department of Oncology, Tongji Hospital of Tongji Medical College, Huazhong University of Science and Technology, Jiefang Road 1095, Wuhan, 430030, China.
3. Cancer Center and Department of Pharmacology and Toxicology, Medical College of Wisconsin, Milwaukee, WI 53226, USA.

# These authors contributed equally

* [Corresponding author](javascript:;)s

Dr. Bin Liu: [liubin@jou.edu.cn](mailto:liubin@jou.edu.cn). Jiangsu Key Laboratory of Marine Pharmaceutical Compound Screening, College of Pharmacy, Jiangsu Ocean University, Lianyungang 222005, China.

Dr. Wei Liu: [weiliu@mcw.edu](mailto:weiliu@mcw.edu). Cancer Center and Department of Pharmacology and Toxicology, Medical College of Wisconsin, Milwaukee, WI 53226, USA.

**Supplementary Figure**


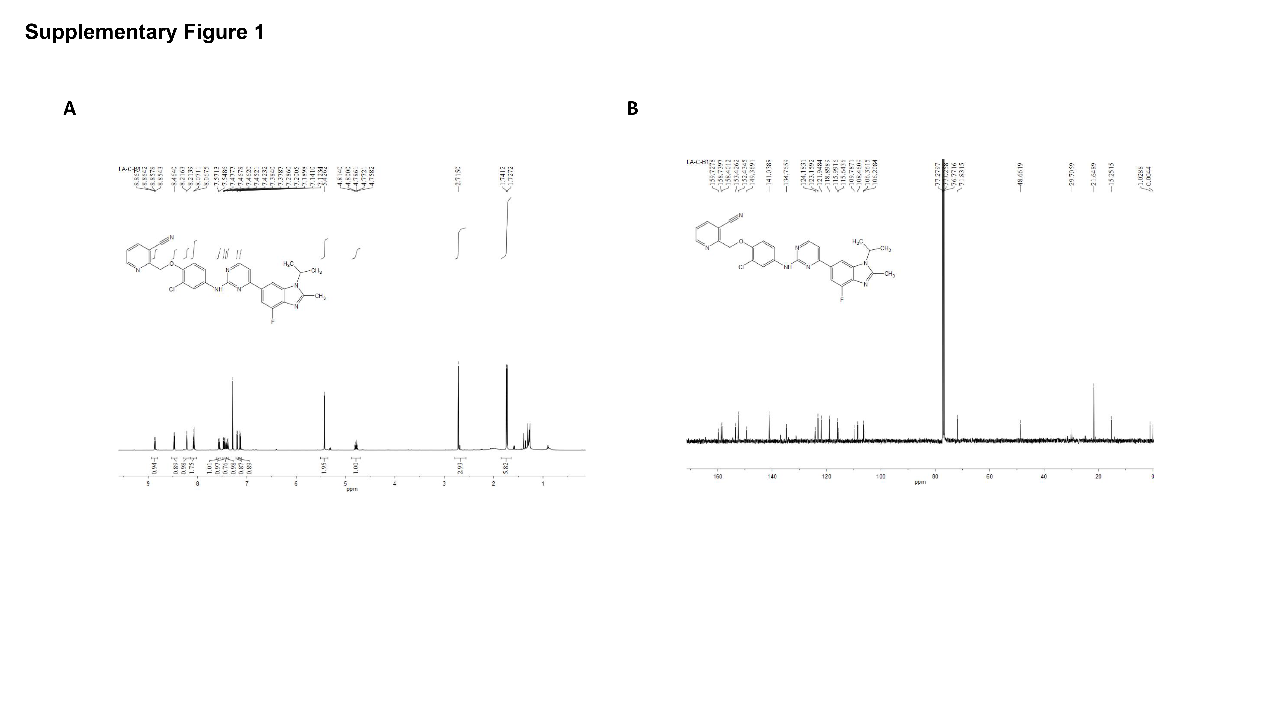
 **Figure S1. Structural Characterization of LA-CB1 by ¹H NMR and ¹³C NMR Spectroscopy.**
(A) ¹H NMR spectrum of LA-CB1 recorded in DMSO-d6. The spectrum shows characteristic proton resonances corresponding to different regions of the LA-CB1 molecule. Peaks between 6-9 ppm correspond to aromatic protons from the benzoimidazole and pyrimidine moieties, while peaks between 1-2 ppm represent the isopropyl and methyl groups. These chemical shifts confirm the expected structure of LA-CB1. (B) ¹³C NMR spectrum of LA-CB1 recorded in DMSO-d6. The spectrum shows carbon resonances corresponding to the aromatic carbons between 120-160 ppm, along with aliphatic carbon signals between 10-40 ppm. The strong peak at 77 ppm is attributed to the residual DMSO-d6 solvent. The spectra confirm the successful synthesis and structural integrity of LA-CB1, consistent with its proposed chemical structure.


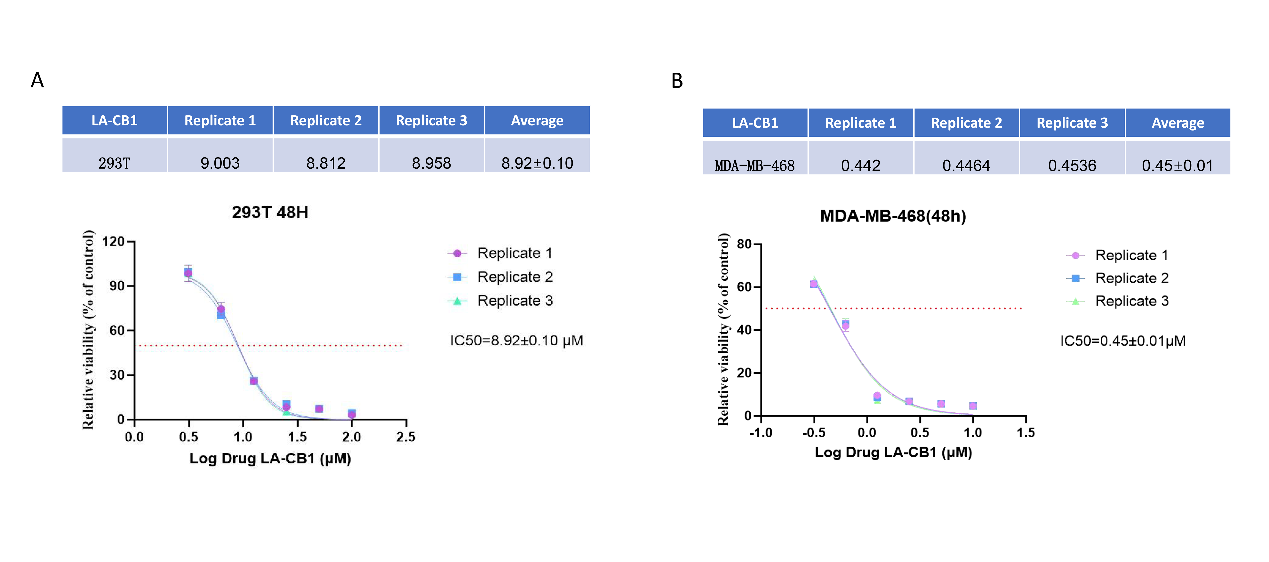


**Figure S2. IC50 of LA-CB1 in 293T and MDA-MB-468 cells, Related to Figure 2.**
(A, B) IC50 values of LA-CB1 on 293T and MDA-MB-468 cell lines determined by MTT assay after 48 hours of treatment. Data are expressed as mean ± SD of three independent experiments.


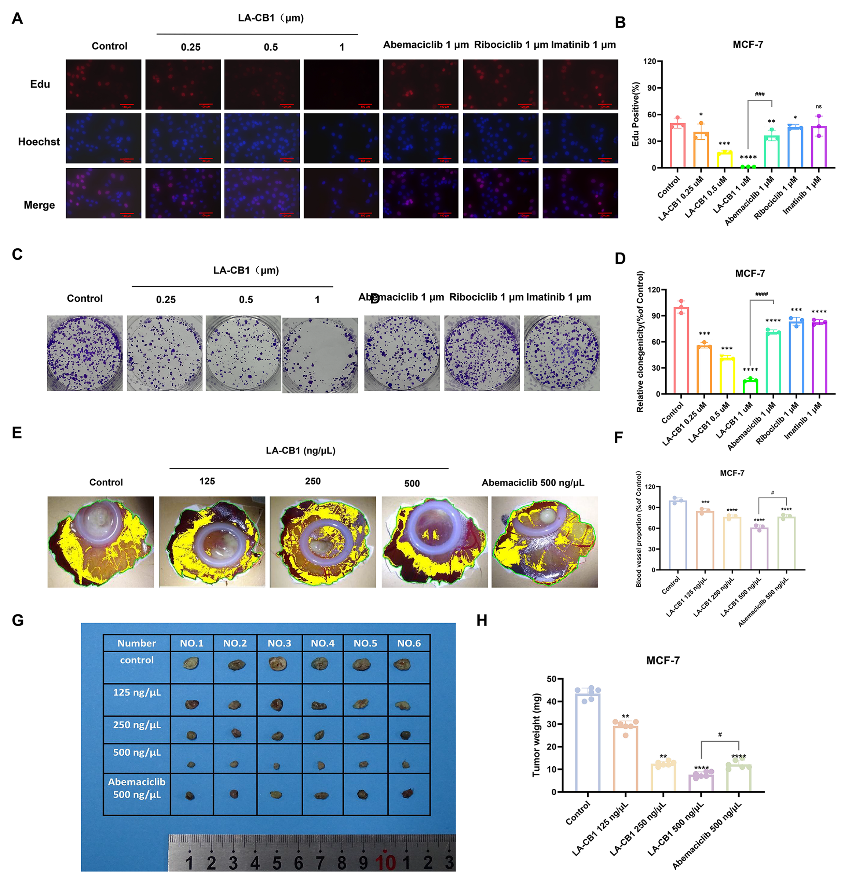


**Figure S3. LA-CB1 Suppresses DNA Synthesis, Clonogenic Growth, and Angiogenesis in MCF-7 Cells, Related to Figure 2.**
(A) EdU incorporation assay in MCF-7 cells. Cells were treated with increasing concentrations of LA-CB1 (0.25, 0.5, 1 µM) for 48 hours, followed by EdU staining. Representative images show EdU-positive cells (red) and Hoechst-stained nuclei (blue). Scale bar, 50 µm. (B) Quantification of EdU-positive cells in MCF-7 cell lines. LA-CB1 significantly reduced EdU incorporation in a dose-dependent manner. Data are presented as mean ± SD (n = 3). p < 0.001 compared to control. (C) Colony formation assay in MCF-7 cells treated with LA-CB1 for 10 days. Representative images of colonies stained with crystal violet. (D) Quantification of relative colony numbers in MCF-7 cells. LA-CB1 markedly reduced colony formation, with 1 µM LA-CB1 inhibiting clonogenic survival by more than 80%. Data are shown as mean ± SD (n = 3). p < 0.001 compared to control. (E) Representative images from the chick chorioallantoic membrane (CAM) assay showing tumor size and vascularization in MCF-7 tumor models treated with increasing concentrations of LA-CB1. (F) Quantification of basal vascular proportion surrounding the tumors in the CAM assay. LA-CB1 significantly inhibited angiogenesis in a dose-dependent manner, with effects comparable to Abemaciclib. Data are represented as mean ± SD (n = 3). p < 0.01 compared to control. (G, H) Measurement of tumor weight in MCF-7 tumors excised from CAM models. LA-CB1 reduced tumor weight in a dose-dependent manner. p < 0.001 compared to control.


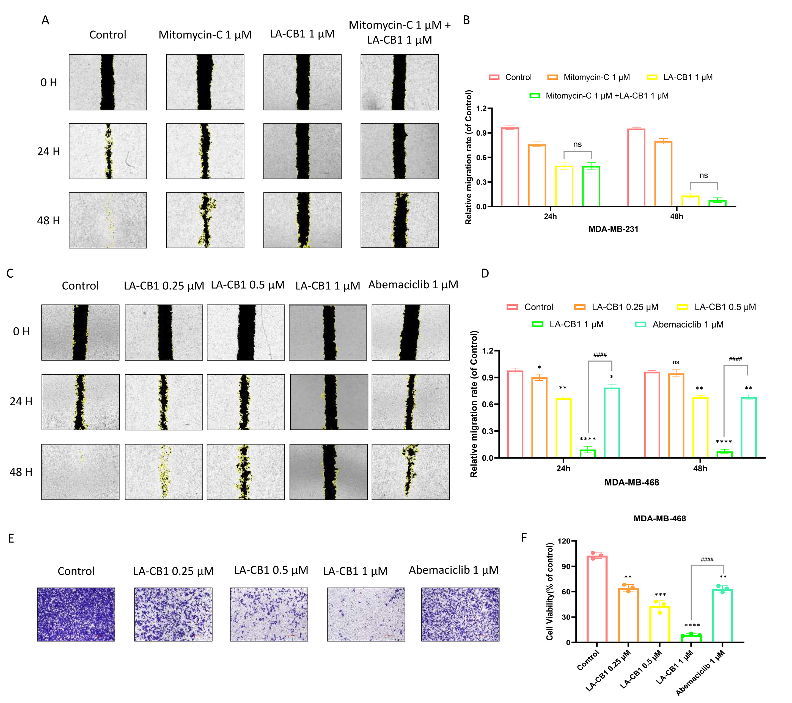


**Figure S4.** **Mechanisms of LA-CB1-Induced Inhibition of Cell Migration and Invasion in Triple-Negative Breast Cancer Cells , Related to Figure 3.**

(A，B)Wound healing assay of MDA-MB-231 cells treated with DMSO, Mitomycin-C 1 μM, LA-CB1 1 μM, Mitomycin-C 1 μΜ +LA-CB1 1 μM respectively. Representative images show wound closure at 0, 24 and 48 hours after treatment. (C，D) Wound healing assay of MDA-MB-468 cells treated with DMSO, LA-CB1 0.25 μM, LA-CB1 1 μM , LA-CB1 1 μM ，Abemaciclib 1 μM respectively. Representative images show wound closure at 0, 24 and 48 hours after treatment. (E，F) Transwell invasion assay showing MDA-MB-468 cells treated with LA-CB1 for 24 hours. Representative images of invaded cells stained with crystal violet.


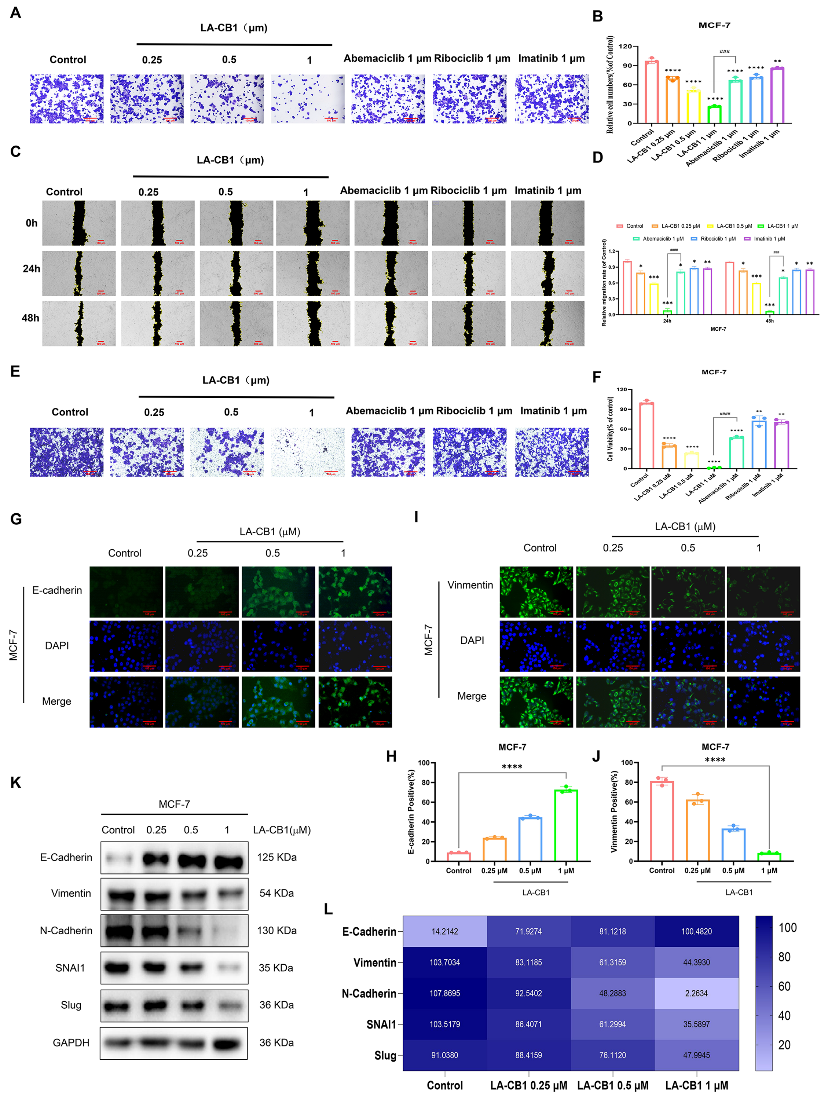


**Figure S5. LA-CB1 Inhibits Cell Adhesion, Migration, and Invasion in MCF-7 Cells, and Modulates EMT Markers, Related to Figure 3.**

(A) Representative images of cell adhesion assays in MCF-7 cells treated with LA-CB1 (0.25, 0.5, 1 µM) for 2 hours. Adherent cells were stained with crystal violet. (B) Quantification of adherent cells following LA-CB1 treatment. LA-CB1 significantly inhibited cell adhesion in a dose-dependent manner. Data are presented as mean ± SD (n = 3). p < 0.001 compared to control. (C) Wound healing assay in MCF-7 cells treated with increasing concentrations of LA-CB1. Representative images show wound closure at 0, 24, and 48 hours after treatment. (D) Quantification of wound closure after 24 and 48 hours of treatment. LA-CB1 significantly delayed wound closure in a dose-dependent manner. Data are represented as mean ± SD (n = 3). p < 0.001 compared to control. (E) Transwell invasion assay showing MCF-7 cells treated with LA-CB1 for 24 hours. Representative images of invaded cells stained with crystal violet. (F) Quantification of invaded cells in the Transwell assay. LA-CB1 significantly inhibited cell invasion in a dose-dependent manner. Data are shown as mean ± SD (n = 3). p < 0.001 compared to control. (G, J) Immunofluorescence staining of E-cadherin (G) and Vimentin (H) in MCF-7 cells treated with increasing concentrations of LA-CB1 for 48 hours. Scale bar, 100 µm. Data are shown as mean ± SD (n = 3). p < 0.001 compared to control. (K, L) Western blot analysis and quantification of E-cadherin, Vimentin, N-cadherin, SNAI1, and Slug expression levels following LA-CB1 treatment. LA-CB1 treatment resulted in a dose-dependent increase in E-cadherin expression and a decrease in mesenchymal markers, including Vimentin and N-cadherin, in MCF-7 cells. Data are presented as mean ± SD from three independent experiments. p < 0.01 compared to control.


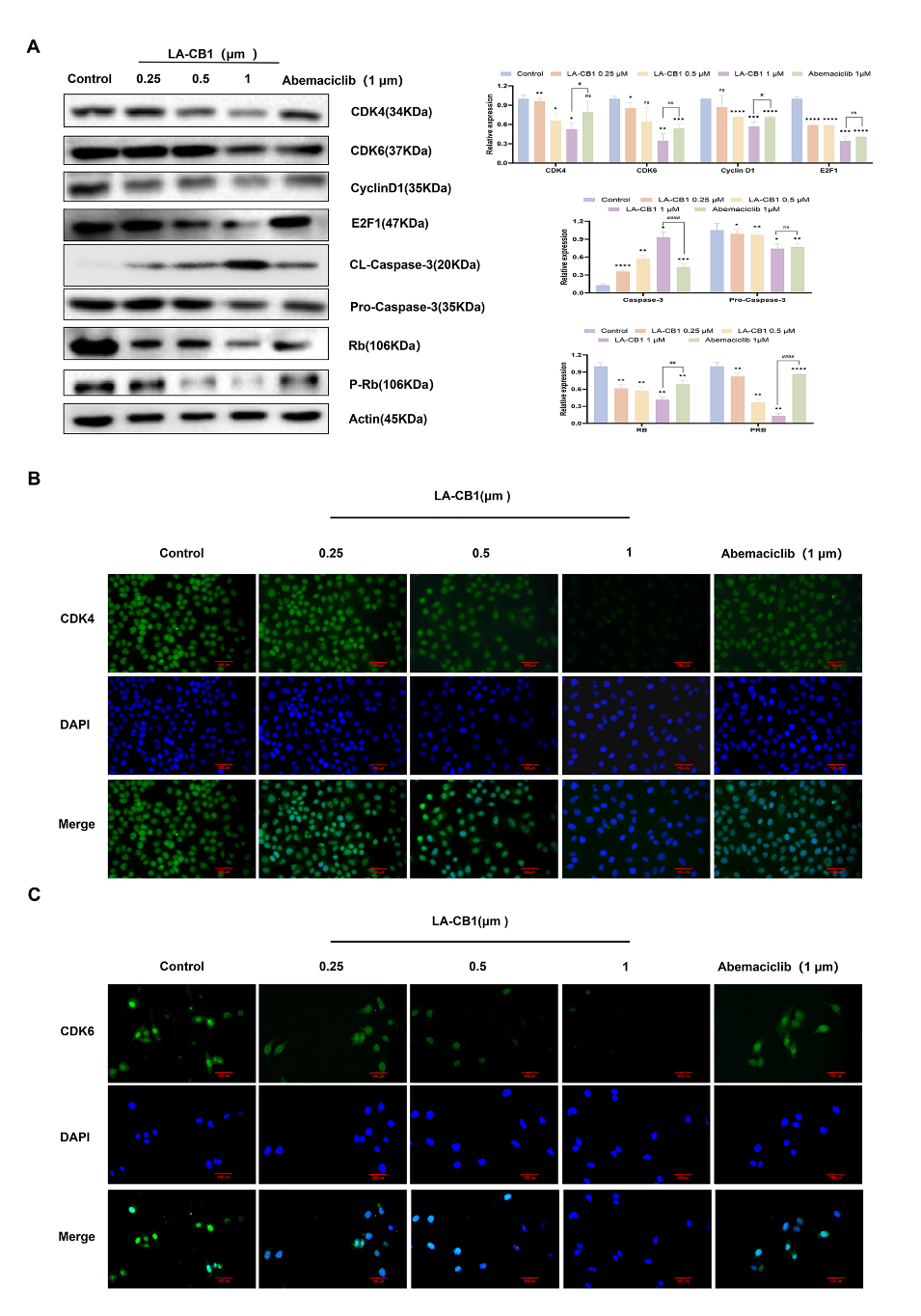


**Figure S6. LA-CB1 Downregulates CDK4/6 and Cyclin D1, and Promotes Caspase-Dependent Apoptosis in MCF-7 Cells, Related to Figure 6.**
(A) Western blot analysis of CDK4, CDK6, Cyclin D1, E2F1, Rb, and cleaved caspase-3 in MCF-7 cells treated with LA-CB1 (0.25, 0.5, 1 µM) for 48 hours. LA-CB1 treatment resulted in a dose-dependent downregulation of cell cycle regulators, including CDK4, CDK6, and Cyclin D1, as well as activation of caspase-3, indicating the induction of apoptosis. (B, C) Immunofluorescence analysis of CDK4 (B) and CDK6 (C) in MCF-7 cells treated with increasing concentrations of LA-CB1. Fluorescence intensity decreased in a dose-dependent manner following treatment with LA-CB1, indicating a reduction in CDK4/6 expression. Scale bar, 100 µm.
